# Graphene-Based Plasmonic Antenna for Advancing Nano-Scale Sensors

**DOI:** 10.3390/nano15120943

**Published:** 2025-06-18

**Authors:** Waqas Ahmad, Yihuan Wang, Guangqing Du, Qing Yang, Feng Chen

**Affiliations:** 1 State Key Laboratory for Manufacturing System Engineering and Shaanxi Key Laboratory of Photonics Technology for Information, School of Electronic Science and Engineering, Xi’an Jiaotong University, Xi’an 710049, China; marralwaqas@stu.xjtu.edu.cn (W.A.);; 2 School of Instrument Science and Technology, Xi’an Jiaotong University, Xi’an 710049, China

**Keywords:** hybrid nanomaterials, plasmonic sensitivity, biomolecular interactions, next-generation biosensing

## Abstract

The integration of two-dimensional graphene with gold nanostructures has significantly advanced surface plasmon resonance (SPR)-based optical biosensors, due to graphene’s exceptional optical, electronic, and surface properties. This review examines recent developments in graphene-based hybrid nanomaterials designed to enhance SPR sensor performance. The synergistic combination of graphene and other functional materials enables superior plasmonic sensitivity, improves biomolecular interaction, and enhances signal transduction. Key focus areas include the fundamental principle of graphene-enhanced SPR, the functional advantages of graphene hybrid platforms, and their recent applications in detecting biomolecules, disease biomarkers, and pathogens. Finally, current limitations and potential future perspectives are discussed, highlighting the transformative potential of these hybrid nanomaterials in next-generation optical biosensing

## 1. Introduction

An optical technique enables real-time, label-free detection of biomolecular interactions to improve the sensitivity of conventional Surface Plasmon resonance (SPR)-based biosensors [1,2]. The scientific observations of light diffraction patterns from metal gratings during the early 20th century [3,4] later recognized this phenomenon as collective oscillations of conduction electrons (surface plasmons) coupled to incident light at metal–dielectric interfaces [5]. The current SPR technology is limited in sensitivity, especially for low-abundance analytes or single-molecule detection. To overcome these challenges, it is necessary to design and synthesize new nanostructured materials that can deliver better coupling between incident light and the analyte. Among these innovations, plasmonic nanoantennas have gained significant attention [6]. Advances in the past decade in designs of nanoantennas, including dipole, bowtie, and slit-based geometries, have demonstrated better performance in biosensing and label-free detection of proteins, nucleic acids, and pathogens [7,8,9]. Their geometry allows precise confinement of localized SPR near the sensing surface of the biosensor, facilitating the detection of subtle refractive index changes associated with biomolecular binding events.

For instance, the role of femtosecond laser-excited plasmonic modes in metallic nanostructures and their impact on the modulation of SPR has been studied, opening new routes for real-time sensing applications [10]. Similarly, it was shown that two-slit plasmonic antennas can be explored to study ultrafast dynamics of light–matter interactions and to understand field localization and signal enhancement [11]. These studies highlight the increasing significance of plasmonic antennas as key enablers in high-performance, ultrafast, and highly sensitive sensing systems.

A significant advancement in this field has been the development of hybrid plasmonic antennas, which integrate noble metals with functional materials such as graphene [12]. Graphene, composed of a single layer of carbon atoms arranged in a 2D hexagonal crystal lattice [13], is not only the thinnest and strongest material known, but also a highly tunable plasmonic medium whose optical response can be engineered through interaction, doping, or external biasing. It offers tunable optical properties, exceptional electrical conductivity, and surface functionalization capabilities, enhancing selectivity and enabling dynamic sensing functions [14,15,16]. Graphene-based plasmonic sensors are built on their ability to detect subtle variations at the micro- and nano-scales. Furthermore, mid-infrared (mid-IR) and terahertz (THz) plasmon modes on the graphene surface spectral regions substantially broaden their utility for sensing organic and biomolecules, an application not so easily available for the conventional plasmonic materials [17,18,19]. The electrically tunable SPP wavelength can potentially enable a new segment of plasmonic devices [20]. Most importantly, the properties of graphene SPPs can be dynamically tuned through chemical doping, applying an external electric field, or a magnetic field [21]. This tunable property makes graphene an attractive material for SPP-based high-performance nanodevices.

However, the optical response of graphene is also dependent on its surface state, doping, and interface effects with other dielectric materials, which might limit the interaction of light with it. The integration of graphene with conventional plasmonic nanostructures has demonstrated advantages in both research fields. Plasmonic nanostructures can improve the optical properties of graphene, whereas graphene can modulate the optical response of plasmonic nanoarrays, creating a synergistic effect for sensing applications. Consequently, noble metal superstructure surfaces combined with graphene can be utilized to control light waves accurately and effectively [22]. This capability enables Graphene’s application in fields such as bioimaging [23], drug delivery [24], and biosensors [25].

In nanophotonics, the most dynamically thriving subfield is plasmonics, which makes use of the nanoscale confinement of light by metallic nanostructures to manipulate optical fields [26,27,28,29]. Optical nanoantennas, an important plasmonic element, exhibit resonance frequency shifts in response to changes in their surrounding medium, making them valuable for biosensing [30,31,32]. Traditional optical biosensors are generally bulky, slow, and expensive [33,34,35], leading to the development of miniaturized waveguide-sensor and fiber-optic sensor approaches; however, these may suffer from sensitivity and accuracy issues [36,37]. Infrared spectroscopy, being a label-free and sensitive technique, can have a low signal-to-noise ratio ata nanoscale [37,38]. The plasmonic nanoantenna-based biosensors were developed to overcome these limitations, by using plasmonic nanoantennas, patterned metallic nanostructures on dielectric substrates, that enabl nanofocused optical energy depending on the properties o metalal and substrate [39,40,41]. The graphene sensitivity is also highly geometry-dependent for plasmonic nanoantennas [42,43,44,45,46], where the sensitivity can be significantly enhanced in the arrays of nanoantennas because of collective plasmon excitation and reduced mutual coupling due to the plasmon characteristics of graphene [47]. Recent advances show that graphene-based plasmonic biosensors can be significantly enhanced by tailoring graphene’s electronic environment. A method is intercalation, where atoms or molecules are inserted beneath the graphene layer to modify its carrier density and dielectric response without compromising its structural integrity. This enables dynamic tuning of plasmonic resonance frequencies, particularly in the mid-IR and THz ranges, improving sensitivity for targeted biomolecule detection [48,49]. The fabrication method also impacts performance: exfoliated graphene provides high purity and minimal defects; CVD-grown graphene supports large-scale production; and epitaxial graphene offers uniformity across wafer-scale devices [50,51]. Each type supports specific sensing needs for tunability, sensitivity, or scalability. Understanding these material-dependent properties is essential for designing miniaturized, reconfigurable SPR sensors that exceed the capabilities of conventional metal-only systems.

This review aims to provide a comprehensive overview of recent advancements in graphene-based plasmonic antennas for surface plasmon resonance (SPR) biosensing. We begin by discussing the fundamental principle of SPR and the physical mechanisms of plasmonic field enhancement. We then explore graphene’s optical and electronic properties that make it suited for nanoscale sensing applications. The review primarily focuses on the integration of advanced hybrid graphene–metal nanoantenna geometries for enhancing the performance of surface plasmon resonance (SPR)-based biosensors. Emphasis is placed on the interplay between material properties, biosensing mechanisms, and recent applications in the detection of biomolecules, disease biomarkers, and pathogens. Finally, we address current challenges and future directions in the development of miniaturized, high-performance optical biosensors based on graphene-integrated plasmonic architectures.

## 2. Fundamentals of SPR and Plasmonic Nanoantennas

### 2.1. Fundamentals of Surface Plasmon Resonance (SPR)

Surface plasmon resonance (SPR) is defined as the resonant oscillations of conduction electrons at the interface between a metal and a dielectric, excited by the incidence of p-polarized light under conditions of total internal reflection. This resonance occurs when the momentum of the incident photons is similar to that of surface plasmon polaritons (SPPs) and leads to a distinctive and narrow dip in the intensity of the reflected light. The local refractive index near the sensor surface has an intensive impact on SPR, so SPR could be set up as a sensor that can be used for label-free, real-time detection of biomolecular interactions [52]. The simplest geometry that can be made to support a surface plasmon has a semi-infinite metal with complex permittivity $\varepsilon_{m}=\varepsilon_{m}^{\prime }+i\varepsilon_{m}^{\prime \prime }$ and a dielectric medium with permittivity $\varepsilon_{d}=\varepsilon_{d}^{\prime }+i\varepsilon_{d}^{\prime \prime }$, where $\varepsilon_{j}^{\prime }$ and $\varepsilon_{j}^{\prime \prime }$ are the real and imaginary parts of (j is m or d). Solving Maxwell’s equations with appropriate boundary conditions reveals that the system only possesses a single transverse magnetic (TM) mode, eh: surface plasmon.

The magnetic field vector lies in the plane of the interface and is perpendicular to the direction of wave propagation, while the electric field is perpendicular to the magnetic field.

If the metal occupies the region z<0, and the SPP propagates along the x-axis, the magnetic field intensity $\vec{H_{J}}$ can be described as

(1) $$\;\vec{H_{J}}=\left(0,\;H_{y},\;0\;\right)=\left(0,1,0\right)Aexp\left[-a_{j}z+i\left(\beta_{x}-\omega t\right)\right]$$

where ω is the angular frequency of the incident light, t is time, β is the propagation constant along the interface, and $a_{j}=\sqrt{\beta^{2}-{\left(\omega /c\right)}^{2}\varepsilon_{j}}$ is the decay constant in the medium, where j = m (metal) or d (dielectric) [53]. The propagation constant $\beta_{SP}$ of a surface plasmon at a metal–dielectric interface is given by

(2) $$\;\beta_{SP}=\frac{\omega }{c}\sqrt{\frac{\varepsilon_{d}\varepsilon_{m}}{\varepsilon_{d}+\varepsilon_{m}}}=\frac{2\pi }{\lambda }\sqrt{\frac{\varepsilon_{d}\varepsilon_{m}}{\varepsilon_{d}+\varepsilon_{m}}}$$

**Figure 1:**
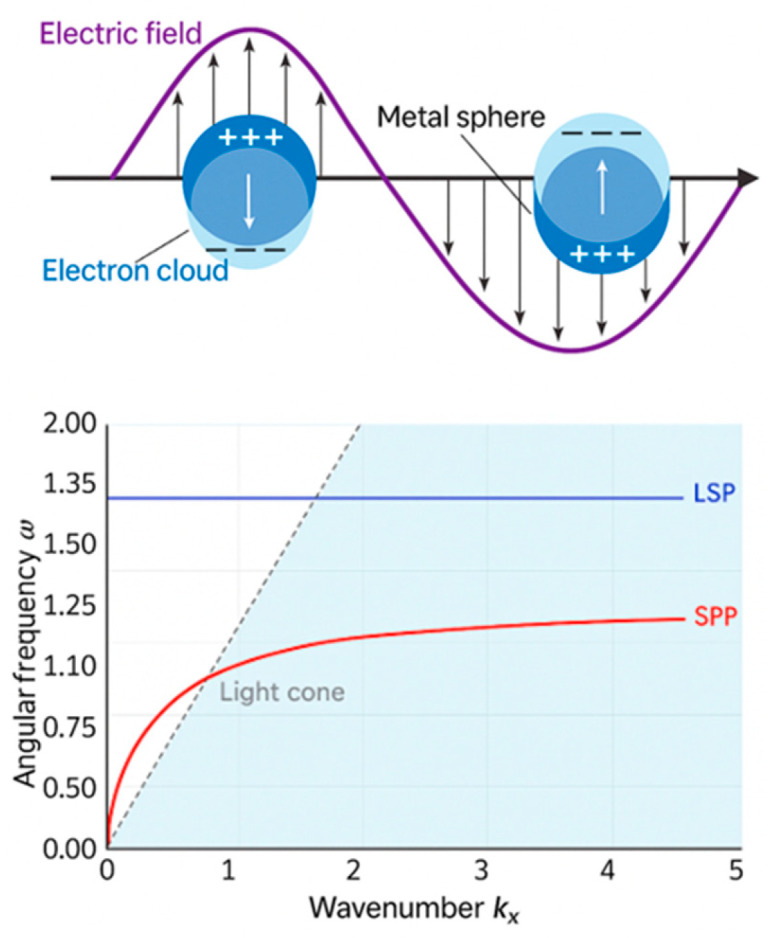
(**top**) Schematic illustration of localized surface plasmon (LSP) oscillations in a metal nanoparticle, where the electron cloud resonates with the incident electric field. (**Bottom**) Lossless dispersion curve for surface plasmon resonance (SPR). The red curve represents the dispersion of SPPs at a metal–dielectric interface, asymptotically approaching the light line. The flat blue line indicates the non-dispersive resonance frequency of LSPs. The light cone represents freely propagating photons in the dielectric [52].

Here, c is the speed of light in a vacuum and λ is the wavelength in vacuum. In a lossless system $\left(\varepsilon_{m}^{\prime \prime }=\varepsilon_{d}^{\prime \prime }=0\right)$, this guide mode can only exit when the real parts of the partitivities are of opposite signs, with the condition $\varepsilon_{m}^{\prime }<-\varepsilon_{d}^{\prime }$ being satisfied. This requirement explains why metals such as gold are commonly used in SPR systems; they provide the negative real permittivity needed to sustain surface plasmons [54,55]. Figure 1 contrasts the behaviors of localized surface plasmons (LSPs) and propagating surface plasmon polaritons (SPPs). While LSPs are confined to nanoparticles and exhibit fixed resonance frequencies, SPPs propagate along metal–dielectric interfaces with a dispersion relationship that deviates from the light line.

One of the main advantages of using graphene for plasmonic nanoantennas is its capabilitto sustainng SPPs of very short wavelengths compared with the free-space wavelengths [56,57]. The following equation gives the dispersion relation describing graphene SPPs:

(3) $$\;q_{p}=\frac{2\pi }{\lambda_{p}}\propto \sqrt{\frac{{\mathrm{e}}^{2}E_{F}}{\hbar^{2}\varepsilon_{0}\left(\varepsilon_{1}+\varepsilon_{2}\right)}}$$

where $q_{p}$ is the plasmon wavevector, $\lambda_{p}$ is the plasmon wavelength, $E_{F}$ is the tunable Fermi energy, ℏ is the reduced Planck constant, and $\varepsilon_{1}$, $\varepsilon_{2}$ are the dielectric constants of the surrounding media [58,59,60]. This relation shows how a higher Fermi energy leads to shorter wavelengths, enabling miniaturized nanoantennas.

Graphene nanoantennas behave like Fabry-Pérot resonators, with a resonance governed by

(4) $$\;2L=m\lambda p=m\frac{2\pi }{q_{p}},\;m=1,2,3,\ldots$$

Here, L represents the length of the antenna, and m refers to the mode number [58,60]. By adjusti Fermi levelng Fermi level $E_{F}$ via electrical gating or chemical doping, one can dy the optical propertiesamically tune the optical properties $q_{p}$, and onsequently the resonance wavelength λp, allowing real-time reconfigurability of the sensor, an attribute unprecedented in traditional metal-based antennas [57,61].

The efficiency of graphene also depends on its complex surface conductivity, as described by the Kubo formula [60]

(5) $$\sigma \left(\omega ,E_{F}\right)={\tilde{\sigma }}_{intra}\left(\sigma \left(\omega ,E_{F}\right)\right)+{\tilde{\sigma }}_{inter}\sigma \left(\omega ,E_{F}\right)$$

This conductivity determines both plasmon propagation lengths and damping, making graphene especially suited for tunable, high-efficiency biosensing applications [60].

**Figure 2:**
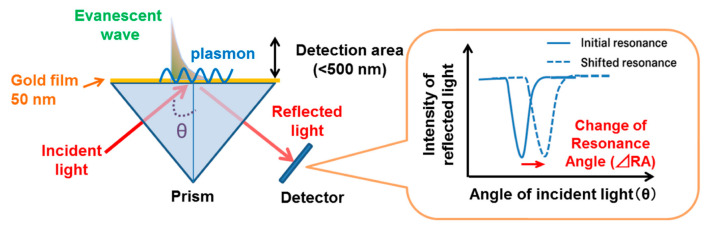
Schematic showing the electromagnetic field distribution at a metal–dielectric interface in SPR sensors. The evanescent field decays exponentially into both materials, with a typical 200–500 nm penetration depth. This near-field interaction governs the sensitivity of SPR to local refractive index changes and results in measurable shifts in the resonance angle [62].

The magnetic field is maximally confined at the interface and decays into both the metal and dielectric, as illustrated in Figure 2. This decay defines the penetration depth, the distance from the interface where the field drops to 1/*e* of its surface amplitude.

Configurations such as the Kretschmann–Raether geometry are commonly used to excite surface plasmons experimentally. In this method, a thin metal film is deposited on the base of a high-refractive-index prism. The p-polarized light is incident at an angle *θ* to generate an evanescent wave. At a specific angle, this wave resonantly couples to the SPP mode when the resonance condition is met, resulting in the appearance of a sharp dip in reflectance.

**Figure 3:**
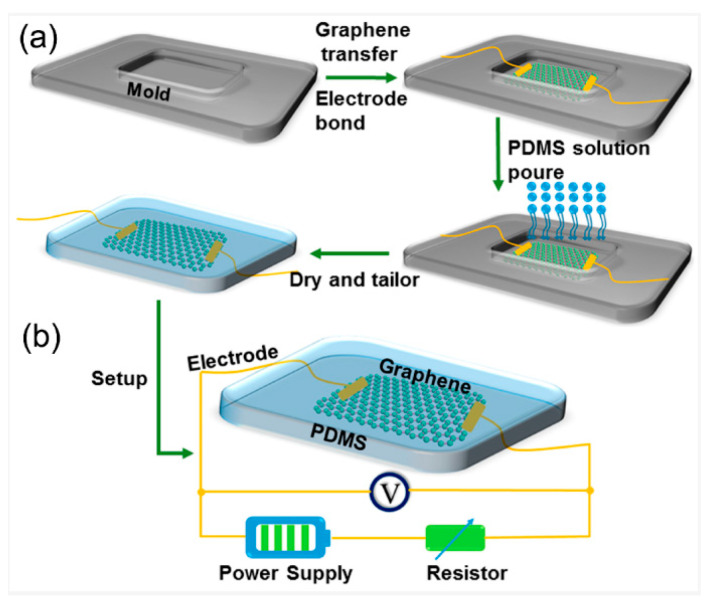
(**a**) Schematic illustration of the fabrication process for the graphene-PDMS (polydimethylsiloxane) flexible device. The process begins with a mold and electrode bonding, followed by graphene transfer and pouring of the PDMS solution. After curing and drying, the device is tailored into the final structure. (**b**) Electrical setup of the fabricated device showing the integrated graphene layer with gold electrodes embedded in PDMS, connected to a power supply and resistor for measurement [63].

The fabrication and electrical setup of the graphene-based PDMS device are illustrated in Figure 3. The process begins with the bonding of electrodes to a mold substrate, followed by the transfer of a graphene layer onto the electrode surface. A PDMS solution is then poured over the setup, encapsulating the graphene and electrode components. After curing, the composite is tailored into its final flexible form. The resulting structure integrates graphene between two electrodes embedded in the PDMS matrix, forming a functional sensing interface. As shown in the schematic, the electrical configuration involves connecting the device to a power supply and a series resistor, enabling voltage measurements across the graphene film for sensing applications.

Surface plasmons are electromagnetic modes localized at metal–dielectric interfaces, typically sustained by waveguide-like structures. The modes are characterized by their respective field distributions and complex propagation constants, which can be derived by solving eigenvalue equations associated with th modes. Notably, the surface plasmons propagation constant is highly sensitive to local refractive index variation. This sensitivity can be quantitatively described using perturbation theory, thereby rendering surface plasmons extremely useful for sensing applications. In practical biosensing applications, the advancement in SPR biosensor technology has significantly improved sensitivity. For instance, conventional SPR sensors utilizing double-tooth ring cavity designs have achieved sensitivities up to 4137 nm per refractive index unit (RIU) [64]. Incorporating graphene and its derivatives has further enhanced sensor performance. A graphene oxide-based SPR sensor demonstrated a sensitivity of 202.2 nm/RIU [65], while a graphene-plasmonic biosensor designed for hemoglobin detection achieved 570 nm/RIU [66]. Moreover, a hybrid configuration combining graphene with other materials has reached sensitivities as high as 10,758 nm/RIU [64]. These developments underscore the significant role of graphene and hybrid materials in advancing SPR biosensor sensitivity.

### 2.2. Plasmonic Nanoantennas: Principles and Types

Plasmonic nanoantennas are sub-wavelength metallic structures desig enhancing electromagneticconfining and enhaenhancectromagnetic fields by excitiand ng localized surface plasmon (LSPs), non-propagating charge density oscillations confined to the nanostructure. Unlike propagating surface plasmon resonance (SPR) in planar films, LSPs generate intense near-fields localized at sharp structures, such as tips, edges, and nanogaps, typically several orders of magnitude stronger than the incident light field [67,68,69,70,71,72,73]. This strong field confinement increases the interaction between the sensing surface and target analyte biomolecule, thereby improving the sensitivity of label-free SPR biosensors. Furthermore, the resonance condition of nanoantennas is governed by their geometry, material composition, and dielectric environment variations, enabling precise tuning of spectral response and spatial field localization. As a result, plasmonic nanoantennas offer a highly versatile platform for high-resolution, real-time biosensing, particularly for the detection of low-abundance analytes [74]. This property makes the use of nanoantennas a powerful tool in biosensing, capable of delivering high field confinement, improved sensitivity, and spatial resolution at the nanoscale. Several nanoantenna designs have proven extremely efficient for SPR-based sensing platforms due to their ability to confine electromagnetic fields and enhance light–matter interactions [75]. Among these, dipole nanoantennas are the simplest structure, generally two linear metallic rods separated by a nanometer-sized gap. Their resonance frequency depends upon the length as well as the surrounding dielectric. All of these structures are widely used for enhancing optical signals such as surface-enhanced Raman scattering (SERS) and fluorescence [76]. Bowtie nanoantennas are two triangular metallic elements placed opposite each other with a narrow nanogap at the intersection, which offer significantly enhanced electric field localization. This geometry enables strong light confinement as well as supports applications requiring high sensitivity performance, such as single-molecule detection and real-time biomolecular interaction analysis. The bowtie structures with sharp corners and small gap sizes are ideal for boosting the SPR signal strength [77]. Another class, slit and aperture antennas, consists of sub-wavelength apertures such as narrow milled slits within metallic films. These geometries take advantage of the principle of extraordinary optical transmission (EOT), where incident light couples to surface plasmons as well as transmits through the nanostructure with intensity exceeding classical predictions. Such designs benefit integrated photonic platforms and biosensing schemes requiring enhanced throughput [78].

**Figure 4:**
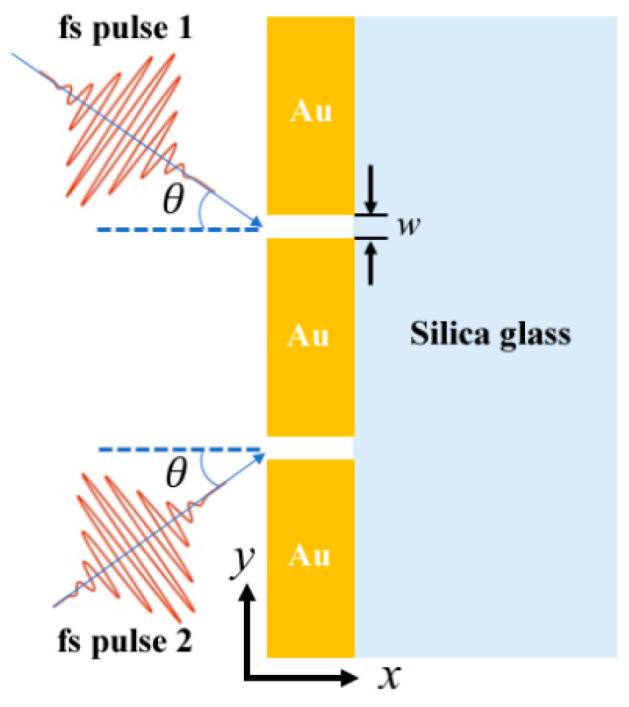
Schematic of the two-slit plasmonic antenna milled in an Au film on top of a silica substrate, excited by a crossed femtosecond laser dual-beam [11].

Figure 4 illustrates the schematic configuration of a dual femtosecond (fs) pulse excitation setup used to investigate plasmonic field enhancement in a nanogap structure. Two fs pulses (Pulse 1 and Pulse 2) are incident at an angle (θ) onto a gold (Au) nanogap patterned on a silica glass substrate. The gap width (**w**) and spatial orientation of the pulses enable strong localized surface plasmon excitation within the Au structures, making this configuration suitable for ultrafast plasmonic switching and high-sensitivity biosensing applications [11].

In addition, the introduction of hybrid as well as multiresonant nanoantennas extended the range of capabilities of plasmonic devices. These nanoantennas combine metals (e.g., Au, Ag) with functional two-dimensional (2D) materials like graphene, thereby combining the field-enhancing properties of metals with the tunability and surface chemistry advantages of 2D materials. Hybridization with graphene allows for dynamic modulation of the plasmonic response, improved biocompatibility, and greater molecular selectivity, all of which are desirable traits in advanced biosensors [79]. Table 1 presents a comparative overview of key plasmonic nanoantenna designs employed in SPR biosensing, highlighting their structural configurations, tunable parameters, and achieved sensitivity. The table summarizes how different geometries, such as dipole, bowtie, slit-based, and hybrid nanoantennas, affect electromagnetic field confinement and biosensing performance. This comparison provides a foundational understanding of how design choices influence sensing efficiency, resolution, and suitability for various diagnostic applications.

Together, these plasmonic nanoantennas, governed by strong near-field localization and geometry-dependent LSPR modes, are instrumental in enhancing the sensitivity of the SPR biosensors. Their field confinement, tunability, and scalable integration are foundational elements for next-generation biosensors. When integrated with graphene and other tunable materials, they extend detection capabilities into mid-IR and THz domains, crucial for label-free, ultra-sensitive molecular diagnostics.

### 2.3. Integration of Plasmonic Nanoantennas into SPR Systems

**Figure 5:**
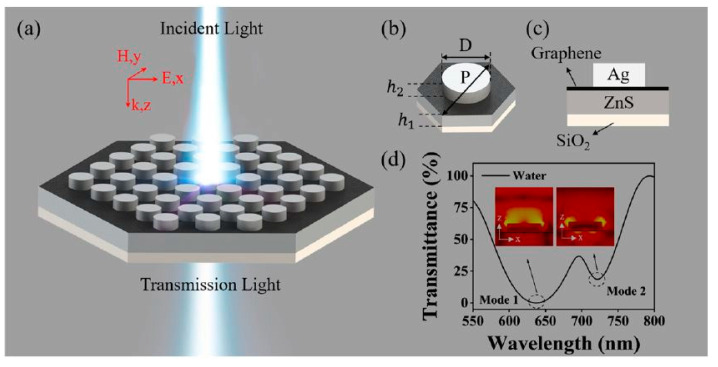
(**a**) Schematic drawing of the LSPR sensing structure. (**b**) Hexagonal periodic dielectric-metal hybrid structure. (**c**) Structural side view. (**d**) The corresponding simulated transmission spectra in water with D = 300 nm, P = 450 nm, h_1_ = 120 nm, h_2_ = 80 nm, and Ng = 20, at normal incidence under TM polarization. The inset of the figure provides electric-field profiles corresponding to the dips [83].

The integration of plasmonic nanoantennas into SPR architectures has significantly advanced the sensitivity, selectivity, and miniaturization of modern biosensing systems. Various configurations have been developed to optimize this integration, depending on sensing environments and performance requirements. For example, on-chip SPR sensors with nanoantenna arrays provide compact designs and compatibility with microfluidic platforms, enabling real-time, multiplexed detection in point-of-care diagnostics. However, they are often limited by fabrication complexity and potential waveguide losses [80]. In contrast, Kretschmann-based SPR systems enhanced by nanoantenna arrays deposited atop metal films combine the benefits of propagating and localized surface plasmon modes, offering high sensitivity and broader spectral tunability. Yet, these setups may suffer from limited reusability and the need for precise angular alignment [81]. A third approach involves waveguide-integrated nanoantennas, which enable efficient light coupling with compact geometries, making them suitable for integrated photonic circuits. However, this demands precise reflective index matching and exhibits limited surface interaction volume [82]. Among the most recent trends are graphene-based hybrid nanoantenna structures, which offer improved sensitivity and dual-channel detection.cbilities in the vis graphenespectrum. This is enabled by grapassis-strengthens field enhancement which strengtheocalizessanalyte mon excitation and improves analyte interaction efficiency [83]. The structural design and optical response of a hybrid graphene–metal plasmonic biosensor are illustrated in Figure 5. As shown in panel (a), the device consists of a periodic hexagonal array of dielectric-metal hybrid structures exposed to incident light, enabling strong plasmonic coupling. Panel (b) defines the key geometric parameters, including the nanopillar diameter D and the heights h_1_ and h_2_ of the constituent layers. The cross-sectional view in panel (c) shows the stacked composition of graphene, silver (Ag), ZnS, and the SiO_2_ substrate, which collectively facilitate enhanced light–matter interaction. Panel (d) presents the transmittance spectrum under water immersion, revealing two prominent resonance dips (mode 1 and mode 2) that correspond to distinct plasmonic modes. The images display electric field distributions at these resonance wavelengths, confirming stronger field confinement near the nanopillars. This configuration plays a critical role in achieving high sensitivity and tunability for biosensing applications, particularly in aqueous environments [83].

Table 2 provides a comprehensive comparison of key SPR integration strategies with plasmonic nanoantennas, outlining their respective advantages and limitations. These include on-chip, Kretschmann, waveguide-integrated, and graphene-hybrid configurations, each offering trade-offs in terms of sensitivity, scalability, and fabrication complexity.

Together, these systems represent a convergence of nanophononics, materials engineering, and biosensing innovation, paving the way for next-generation SPR platforms capable of ultra-sensitive detection in clinical and point-of-care settings.

### 2.4. Advantages of Graphene in Plasmonic Nanoantennas

Due to its atomically thin form and tunable electronic band structure, graphene exhibits strong light–matter interaction and supports tightly confined surface plasmon modes, making it extremely beneficial for plasmonic nanoantennas for nanoscale sensors [84,85,86]. Its massless Dirac fermion charge carriers are responsible for its exceptional plasmonic performance, having ultrafast mobility of the carrier and linear dispersion of the energy-momentum relationship [87]. All of these features provide a highly tunable electronic structure of graphene for dynamic adjustment of the Fermi energy level using external gating or chemical doping [88]. This tunability directly influences the confinement and propagation of surface plasmon polaritons (SPPs), which are fundamental to the operation of plasmonic nanoantennas.

The relationship demonstrates the plasmon wavelength in graphene $\left(\lambda_{p}\right)$ cattains reductions up to two orders of magnitude compared to the free-space wavelength at the same frequencies, thus allowing tiny miniaturization of antenna elements [89]. A graphene-based THz nanoantenna allows researchers to decrease its physical size by 22 times over a conventional metallic antenna having comparable resonating frequencies [90]. An equally important advantage is the electrical tunability of the plasmonic resonance achieved by gating or doping graphene, which changes the Fermi energy ($E_{F}$) and thus alters the resonant frequency or wavelength [88,91]. The tunability is directly demonstrated in graphene bowtie antennas: by changing the graphene chemical potential, one can realize frequey reconfiguration in a substantially terahertz band [90].

**Figure 6:**
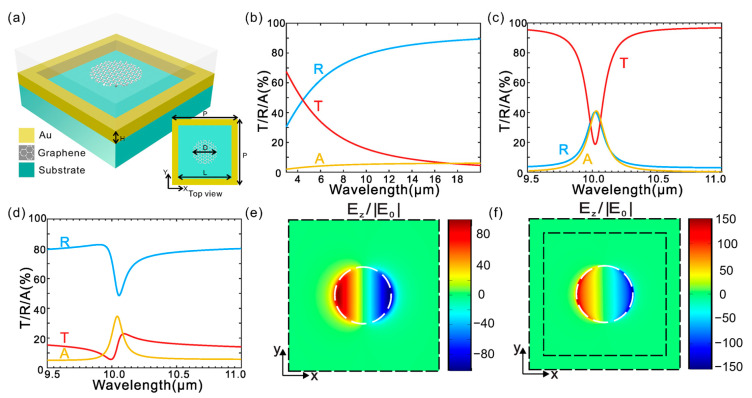
Design and optical response of a hybrid metal-graphene Fano-resonant metamaterial. (**a**) Schematic illustration of the proposed hybrid metamaterial structure. (**b**–**d**) Simulated optical spectra showing transmission, reflection, and absorption for three configurations: (**b**) nanostructured gold film without graphene nano disks, (**c**) graphene nano disks without nanostructured gold, and (**d**) the complete hybrid metamaterial combining both components. (**e**,**f**) distributions of the local electric field in the z-direction at the resonance wavelength for (**e**) graphene nano disks (~10 μm) and (**f**) the hybrid structure (~10.05 μm). The field is normalized to the incident field amplitude E_0_ and plotted in the x-y plane located 5 nm above the graphene layer. An x-polarized plane wave is incident normally from the top side of the structure [93].

Figure 6 illustrates the optical response and field distribution of a graphene-Au plasmonic nanoantenna structure designed for mid-infrared operation. The spectral plots (b–d) demonstrate strong resonance features in transmittance (T), reflectance (R), and absorbance (A), with clear tunability across the 9–11 μm wavelength range. The electric field enhancement maps (e, f) reveal intense near-field localization at the graphene–metal interface, confirming efficient confinement. This configuration leverages graphene’s tunable conductivity and high confinement to achieve reconfigurable plasmonic responses, offering significant advantages for sensing applications requiring narrowband, high-sensitivity mid-IR detection.

Graphene plasmonic antennas have exhibited lower losses than conventional metals [92,93]. This is largely attributed to the fact that electron–phonon scattering rates in graphene are lower than those in metal, which results in longer plasmon propagation lengths and higher radiation efficiencies [92]. For example, graphene nanoantennas have been demonstrated to achieve radiation efficiencies in the range of 5–16% in the fundamental THz plasmonic modes, an impressive result given their sub-wavelength field confinement and compact structure [60]. The highly localized plasmonic fields in graphene can significantly enhance the light–matter interactions at the sensor’s surface [61,94]. This results in large increases in the sensitivity of the system as subtle alterations such as molecular binding or changes in the refractive index near the surface of the graphene induce detectable plasmon resonance shifts [83,91].

Table 3 presents a performance comparison between conventional metal-based plasmonic antennas and graphene plasmonic antennas across key metrics such as field confinement, tunability, miniaturization, and sensitivity. The results highlight graphene’s superior capabilities, including up to 100× λ field confinement, electrical tunability via Fermi level control, and significantly enhanced sensitivity and figure of merit (FOM), making it a promising platform for next-generation reconfigurable and ultra-sensitive biosensors.

Graphene plasmonic resonances in the terahertz to the mid-infrared spectral regime, combined with the vibrational fingerprint frequency of many biomolecules, form a key factor [95,96]. This broad operational bandwidth enables the development of multifunctional sensor platforms, capable of covering multiple important wavelength spectral ranges for gas monitoring and biochemical sensing [97]. Graphene’s planar structure property and its compatibility with dielectric and metallic substrates further allow seamless integration of hybrid plasmonic devices, such as metal-graphene sensors, achieving the dual-band or multi-spectral sensing based on combined Fano resonances together with the tunable graphene plasmonic [98,99,100,101,102,103].

**Scheme 1:**
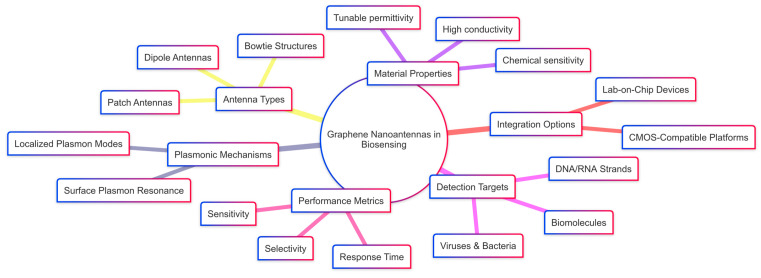
Advantages of graphene-based plasmonic nanoantennas in biosensing applications.

Scheme 1 provides a conceptual map of the key factors influencing the design and application of graphene nanoantennas in biosensing. It outlines the relationship between antenna types, material properties, detection targets, plasmonic mechanisms, integration strategies, and performance metrics. This holistic framework highlights the multifunctional role of graphene-based nanoantennas, emphasizing their tunability, compatibility with lab-on-chip platforms, and potential for highly sensitive, selective, and rapid biomolecular detection.

Advanced designs of graphene antennas successfully achieve 85% radiation efficiency. Research has optimized metrics, including radiation efficiency, gain, and return loss, leading to maximum efficiency combined with directivity values exceeding 8 dB in both simulated and experimental designs [104,105]. This conductivity determines the SPP propagation constant (and thus losses), with higher mobility (long relaxation time, τ) leading to reduced non-radiative damping [90,100]. The external voltage or chemical doping tunes the dispersion and damping of SPPs, thus allowing the design of antennas with customized response and quality factors [106]. Additionally, equivalent circuit models represent the graphene plasmonic nanoantenna as RLC resonators, enabling systematic optimization of field enhancement, resonant frequency, and radiation efficiency by tuning antenna geometries [107].

## 3. Graphene-Based Plasmonic Nanoantennas in Biosensing

### 3.1. Graphene-Gold Hybrid Structures

**Figure 7:**
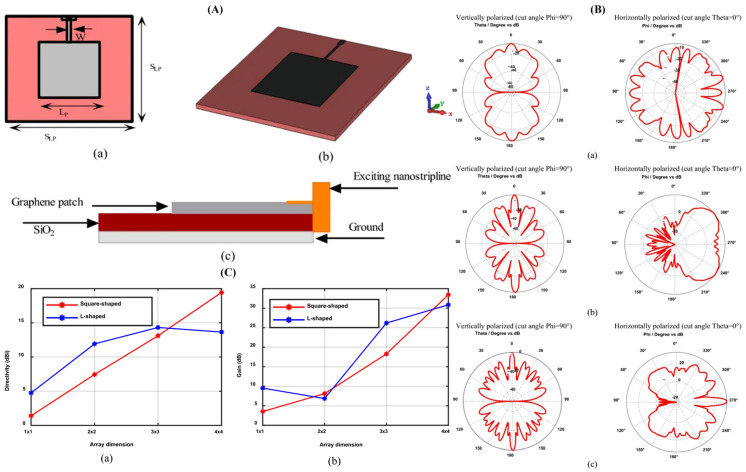
Design, radiation patterns, and performance analysis of graphene-based plasmonic nanoantenna arrays. (**A**) Schematic views of a graphene square patch nanoantenna: (**a**) top view showing patch dimensions and feed width, (**b**) 3D perspective view, and (**c**) side view indicating material layering with SiO_2_ substrate and nanostrip line feed. (**B**) Radiation patterns of graphene nanoantenna arrays for different configurations: (**a**) 2 × 2, (**b**) 3 × 3, and (**c**) 4 × 4 arrays, showing vertically and horizontally polarized field distributions. (**C**) Comparative analysis of directivity (**a**) and gain (**b**) for square-shaped vs. L-shaped graphene nanoantenna arrays across increasing array dimensions [110].

Graphene-based nanoantennas with noble metal plasmonics have emerged as a cutting-edge technology for biosensing due to their exceptional ability to combine graphene’s properties with the plasmonic resonance effect of noble metals such as gold [108]. This integration yields hybrid nanostructures that provide enhanced signal detection, tunability, and sensitivity mechanisms crucial for advanced biosensing applications [109]. This section explores the fine details of the structural components of graphene-gold hybrid nanoantennas, discusses the physical and chemical mechanisms of their signal improvement as well as their enhanced sensitivities, and discusses the advanced fabrication thodologies and smaterials approaches that facilitate their development as well as integration into optimized forms [110]. Graphene-gold hybrid nanostructures offer a class of plasmonic materiwhere ultrathin grane layers are intimately interacting with metallic gold nanostructures to create platforms with enhanced plasmonic and electronic performance functionalities [111]. Owing to its exceptional electrical conductivity, optical tunability, chemical stability, and mechanical strength, graphene serves as an active medium and a structural interface in hybrid biosensing platforms. These attributes enable enhanced field confinement, improved analyte interaction, and integration flexibility in nanoscale sensor architectures [112]. Figure 7 presents the design, simulation, and performance evaluation of a graphene-based patch nanoantenna array. Panel (A) shows a schematic view of a square graphene patch nanoantenna, including (a) a top view highlighting the patch dimensions (S_x_ and S_y_) and feed width, (b) a 3D model showing the antenna integrated on a dielectric substrate, and (c) a side view detecting the material stack with a SiO_2_ substrate and nanostrip line feed. Panel (B) shows simulated far-field radiation patterns for antenna arrays of varying sizes, (a) 2 × 2, (b) 3 × 3, and (c) 4 × 4, demonstrating both vertical and horizontal polarization modes with strong directivity and polarization control. Panel (C) presents a comparative performance analysis: (a) directivity and (b) gain of square-shaped versus L-shaped antenna arrays as a function of array dimension, confirming improved radiation characteristics with increased array size. These results highlight the antenna’s potential for high-efficiency, frequency-reconfigurable applications in terahertz biosensing and plasmonic signal modulation [110].

When gold nanoparticles (AuNPs) are merged with graphene, the resulting hybrid material experiences an interplay between graphene’s delocalized electrons and the localized plasmonic fields of AuNPs [113]. The hybridization significantly enhances the effects of localized surface plasmon resonance (LSPR), manifesting in strong confinement of the electromagnetic field along with higher sensitivity within the visible to near-infrared spectral regime [114,115,116]. For example, the development of freestanding single-layer fabrication of large-area graphene-gold sandwich structures has allowed direct observation of variations in localized electronic structure due to interactions of graphene-Au at the nanoscale, revealed through scanning transmission X-ray microscopy, underpinning the source of enhanced plasmonic activity within these systems [117].

The efficacy of the hybrid structure is further exemplified through graphene stripes integrated with gold gap-ring nanoscale geometries, leading to ultra-narrow LSPR linewidths and resonant intensity enhancements by over two orders of magnitude [118]. These enhancements translate to an impressive sensor sensitivity of approximately 1000 nm/RIU and figures of merit reaching up to 383, which are critical for detecting subtle biomolecular refractive index changes [119]. Additionally, combining metal-graphene hybrid nanoantennas with golden ratio rectangular resonators enables near-perfect infrared absorption, significantly enhancing the intensity of surface-enhanced infrared absorption (SEIRA). This makes for efficient identification for detecting low-concentration explosive molecules, testifying to the practical utility of graphene-Au hybrid materials [120].

Chemically, the decoration of graphene oxide (GO) or reduced graphene oxide (rGO) with AuNPs induces localized plasmons that enable resonance energy transfer mechanisms essential for photonic and electrochemical biosensing applications [121]. The functional groups of the GOs, such as carboxyl, hydroxyl, and epoxy bridges, facilitate covalent, along with non-covalent, interactions with AuNPs, leading to stable nanocomposites that show localized surface plasmon resonance (LSPR) in resonance energy transfer routes for effective biosensing. Such composites are compatible with label-free immunoassays for protein detection from femtomolar levels of protein concentrations, with dynamic signal responses indicating ultra-high selectivity as well as sensitivity and specificity, potentially suitable for point-of-care diagnosis and clinical biomarker sensing purposes [122].

Thus, graphene-gold hybrid structures represent a key element in plasmonic nanoantenna biosensors, leveraging the electronic coupling and morphological tunability of graphene and Au nanomaterials to enhance biosensing performance through stronger electromagnetic confinement and chemical interactions [108].

### 3.2. Mechanisms of Single Enhancement and Sensitivity Improvement

Graphene-based plasmonic biosensors were achieved with high sensitivity and signal enhancement through multiple interrelated physical and chemical mechanisms [123]. A basic mechanism is the coupling of graphene plasmons with localized gold nanostructures-supported surface plasmons. This generates intensified electromagnetic hot spots, nanoscale regions with significantly enhanced electric fields that profoundly boost light-biomolecule interactions with amplifying signal intensity [124,125].

Surface-enhanced Raman scattering (SERS) is epitomized by the most prominent enhancement mechanism of such hybrids [126]. The 2D platform of graphene provides a chemically active surface on which substrate charge transfer interaction of adsorbates is facilitated by its two-dimensional system, a chemical mechanism (CM) supplementing the electromagnetic mechanism (EM) offered by gold nanoparticles. The EM amplifies the intensity of the local optical field while the CM allows Raman signal athe mplification by the resonance through mixing of molecular orbitals and the redistribution of charge [123]. In crumpled three-dimensional graphene-gold hybrid nanostructures, the volumetric increase of plasmonic hotspots enhances local field enhancement to higher values, providing at least one order of magnitude improvement in SERS sensitivity over flat hybrids [127]. The three-dimensionality of these nanostouctures facilitates enhanced access of the analyte and multiple directions of plasmon coupling for optimum biosensing performance. Nonlinear optical processes in graphene provide signal enhancement, as evident from the plasmonic Stokes emission enhancement through resonance [128]. It is a phenomenon involving resonant plasmonic excitation, amplifying inelastic Raman scattering beyond linear absorption resonances, offering large cross-sectioains of vital interest for sensing of low-abundance biomolecules. Inthe mid-infrared spectral range which is important for probing biomolecular vibrational modes, graphene,-based hyperbolic metamaterials (HMMs) and graphene-transition metal dichalcogenide (TMDC)-graphene hybrid metasurfaces have been designed to confine plasmon resonance energy by extreme spatial confinement as well as spectral tunability [129].

**Figure 8:**
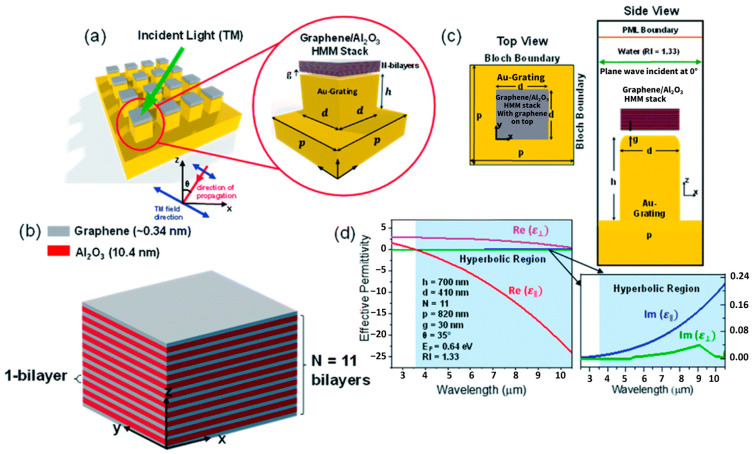
Illustrates a graphene-based hyperbolic metamaterial (HMM) biosensor integrated with a gold grating structure for mid-infrared plasmonic sensing. (**a**) Design overview showing the Au grating coupled with a graphene/Al_2_O_3_ multilayer HMM stack under TM-polarized incident light. (**b**) Three-dimensional model of the unit cell with alternating graphene and Al_2_O_3_ bilayers (N = 11). (**c**) Top and side views highlighting the geometry of the grating and HMM layers. (**d**) Effective permittivity analysis showing real and imaginary components, indicating a hyperbolic dispersion regime beyond 3.58 μm [129].

Figure 8 illustrates a graphene/Al_2_O_3_-based hyperbolic metamaterial (HMM) integrated with a gold grating structure, designed to support tunable mid-infrared plasmonic modes. The multilayer HMM stack enables broadband hyperbolic dispersion, enhancing field confinement and sensitivity, which is advantageous for high-performance SPR biosensing applications.

These hybrid architectures facilitate ultrasensitive label-free biosensing by magnifying the local electromagnetic density of states and the efficiency of light–matter interaction, often by two or more orders of magnitude compared to bare metal substrates. For example, gold-graphene hybrid structures could boost surface field localization [130]. The electronic tunability of graphene plays a pivotal role in plasmonic antenna design. As reported [131], electronic gating and chemical doping tune the Fermi level and carrier concentrsation of graphene, which in turn alter its surface conductivity and plasmon resonance frequency. This tunability allows real-time configurability of the resonance profile, an advantage not found in conventional metallic antennas. All of these mechanisms orchestrate a synergy that enhances biosensing signals by combining intense plasmonic confinement of, the electric field, chemical affinity and charge transfer processes, nonlinear optical effects, and dynamic tuning of plasmon resonance [126].

### 3.3. Fabrication Techniques and Material Design

The fabrication of graphene-based plasmonic nanoantennas requires advanced methodologies for integrating graphene’s delicate two-dimensional lattice into nanoscale metal structures without preserving each material’s functional properties [132]. Several approaches have been developed for the realization of hybrid nanostructures with nanoscale precision of size, shape, and spatial arrangement crucial for plasmonic functionality.

#### 3.3.1. Chemical Synthesis and Surface Functionalization

Chemical synthesis represents a foundational technique in constructing graphene–metal hybrid nanostructures for plasmonic biosensing. Graphene oxide (GO), commonly used in biosensor fabrication due to its rich surface chemistry and aqueous processability, is typically synthesized via the modified Hummers’ method [133]. This technique enables control over flake size and oxygen functional group density, both of which influence the optical response and biocompatibility of GO-based plasmonic devices. Surface functionalization is crucial for bioconjugation. Thiol-gold chemistry is widely used to attach biomolecules onto AuNPs, while amine-carboxyl coupling enables GO binding to peptides, DNA, or antibodies, offering robust, biocompatible interfaces [134,135]. These functionalized nanostructures serve as key platforms for immunoassays, enhancing plasmonic signal specificity.

#### 3.3.2. Self-Assembly and 3D Structural Engineering

Self-assembly techniques introduced scalable fabrication routes that exploit mechanical deformation. For instance, hybrid graphene-gold nanostructures cleverly take advantage of thermally induced shrinkage as well as strain engineering [136]. On substrates that thermally shrink and elastic mismatch, initially planar graphene-AuNP films buckle into crumpled structures, offering expanded plasmonic active volumes and mechanical stability [97]. These 3D structures expand plasmonic active volumes, support increased mechanical stability, and have demonstrated significantly enhanced SERS sensitivities, adopted complex geometries, and extended biosensing applications [137]. Strain-induced assembly also supports integration on flexible and stretchable substrates, opening applications in wearable biosensors. These 3D constructs provide multi-angle light confinement, increasing hot-spot density and sensitivity in label-free SPR platforms.

#### 3.3.3. Lithographic Patterning and Photonic Integration

Photonic device integration involves stacking hybrid waveguides composed of silica (SiO_2_), graphene, and gold layers and optimizing light confinement and absorption. The low-loss dielectric layer between graphene and Au reduces the non-radiative losses, while the patterned nanoscale gold structures act as plasmonic nanoantennas, serving as localized hotspots that enhance field confinement. Integrated electrical contacts enable high-gain performance, wide bandwidth operation, and tunable spectral responses [138]. Advanced techniques such as electron-beam lithography (EBL) and focused-ion beam (FIB) milling remain key techniques for creating graphene bowtie, dipole, and slit nanoantenna structures with nanometer-e precision. These methods enable the precise control over tip sharpness, interparticle gaps, and antenna orientation, all critical for tailoring localized surface plasmon resonance (LSPR) responses [139].

#### 3.3.4. Data-Driven Design and Simulation

Computational techniques, especially finite-difference time-domain (FDTD) simulations, play a critical role in predicting the optoelectronic response of graphene-based plasmonic nanoantennas. These simulations consider the complex dielectric properties of graphene and gold, near-field localization, and the impact of geometry on resonance [139]. Recent advances have introduced deep learning and inverse design frameworks to rapidly optimize antenna shapes for specific spectral performance. These tools reduce time and accelerate material discovery for multifunctional biosensors [140].

**Figure 9:**
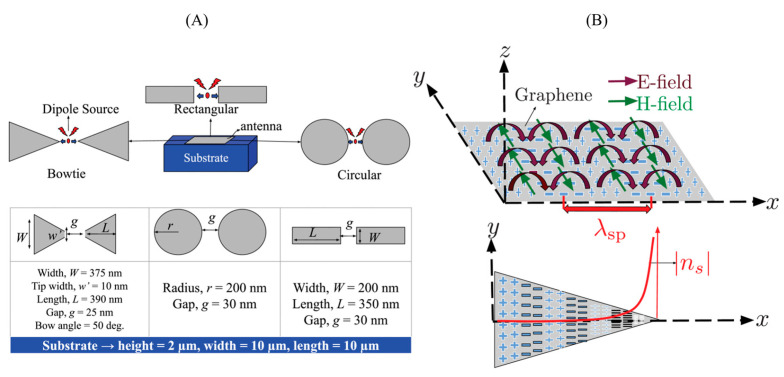
(**A**) Schematic of different plasmonic nanoantenna geometries commonly used in SPR-based biosensing: bowtie, circular, and rectangular antenna pairs positioned on a dielectric substrate. Dimensions for each geometry are annotated, showing width, length, and interparticle gap. (**B**) Conceptual illustration of localized surface plasmon resonance (LSPR) excitation and field distribution on a graphene surface. The electric (E-field) and magnetic (H-field) components of the surface plasmon mode are shown, along with the propagation direction λsp and $\left(\left|n_{s}\right|\right)$ near-field intensity decay [140].

Figure 9 illustrates various graphene-based nanoantenna geometries, including bowtie, rectangular, and circular designs, alongside simulation-driven analysis of electric and magnetic fild confinement. The data-driven modelling demonstrates how geometric parameters directly influence plasmonic wavelength (λ_sp) and near-field enhancement, offering a predictive framework for optimizing biosensor sensitivity through structural tuning and electromagnetic response analysis.

#### 3.3.5. Graphene Integration with Non-Metallic Materials

While noble metals dominate plasmonic antenna designs, combining graphene with non-metallic nanomaterials such as oxides (e.g., ZnO, TiO_2_) and sulfides (e.g., MoS_2_, WS_2_) has emerged as a route for enhancing biosensing functionality. These materials introduce tunable band gaps, high surface area, and strong charge-transfer capabilities. For example, graphene-ZnO hybrids exhibit stronger photonic–plasmonic interactions and increased biomolecule absorption, improving both sensitivity and selectivity in biosensor applications. Similarly, MoS_2_-graphene combinations provide mid-infrared tunability and increased signal-to-noise ratio in label-free platforms [141]. Such heterostructures also demonstrate improved biocompatibility and environmental stability, making them ideal for flexible or wearable diagnostics.

In summary, the fabrication of graphene-based plasmonic nanoantesnna integrates diverse material design in biosensing, chemical synthesis, mechanical assembly, precise lithography, and computational innovation to produce high-performance hybrid systems [142]. As plasmonic biosensing moves toward flexible, miniaturized, and reconfigurable systems, these fabrication techniques offer precise control over optical enhancement, field confinement, and molecular selectivity. Integrating graphene with both metallic and non-metallic materials has opened new design paradigms for next-generation sensors, combining plasmonic tunability with functional versatility.

## 4. Biosensing Applications of Graphene-Based Plasmonic Nanoantennas

### 4.1. Biomolecular Detection

Surface plasmon resonance (SPR) biosensors have emerged as powerful analytical tools capable of investigating biomolecular interactions in real-time without the need for labeling [143,144]. At the core of most SPR biosensors is the label-free detection mechanism, which monitors intrinsic changes in the refractive index at the sensor surface as target molecules bind [145,146]. This approach preserves the native biological activity of the analyte and enables continuous, real-time measurement with high sensitivity, reaching femtomolar levels for some proteins and nucleic acids [147].

In contrast, labeled detection methods employ external signal-generating probs such as enzymes, nanoparticles, fluorescent tags, or radionuclides to amplify the response [148]. While these methods may offer lower detection limits in specific cases, they are generally more time-consuming and complex, and incompatible with real-time analysis due to the need for pre-labeling and multiple sample preparation steps. The choice between label-free and labeled approaches for detection depends on various factors, including the biomolecular compound utilized, analyte type, biological binding site, biosensor design, sample volume, operational cost, analysis time, and required sensitivity [144]. Importantly, the integration of advanced materials like graphene into SPR platforms has significantly enhanced label-free biosensing by increasing the surface area for biomolecule adsorption and improving signal-to-noise ratios through superior electronic properties [144].

Additionally, many SPR mechanisms incorporate electrochemical detection methods such as voltammetry, potentiometry, impedance spectroscopy, and conductometry to improve sensitivity and versatility [149]. For example, Voltametric procedures use redox-active compounds like ferricyanide to produce detectable current upon analyte binding, while impedance procedures track changes in the system’s response to alternating currents, offering details insights into interfacial binding events [149]. Together, these developments highlight the growing distinction and synergy between label-free and labeled detection techniques in modern SPR biosensor design.

### 4.2. Disease Biomarkers and Diagnostics

The detection of disease biomarkers by using SPR biosensors has transformed clinical diagnostics by providing invaluable insight into disease progression and enabling timely intervention [150]. Biomarkers are objective indicators of medical conditions that can be accurately observed from outside the patient and are key to disease diagnostics and monitoring. The molecular indicators are divided into prognostic biomarkers, which provide information about disease recurrence and disease detection using diagnostic biomarkers [151]. Rapid tracking of molecular interactions of SPR technology enables it to be a means of real-time diagnostics [152].

Protein biomarkers are particularly valuable in disease detection due to their presence in various biological fluids and tissues [153]. These biomarkers are expressed differently depending on the disease type, providing diverse information about disorders within the body [154]. For diagnostics of cancer, a variety of protein biomarkers have been identified, such as KRAS for pancreatic cancer, HER_2_ for breast cancer, AFP for liver cancer, and PSA for prostate cancer [155]. The detection of these cancer biomarkers using SPR biosensors facilitates early diagnostics and improves treatment strategies [156]. For the diagnostics of cardiovascular disease, SPR biosensors have also been successfully used to detect biomarkers such as C-reactive proteins (CRP) and S100 beta proteins [157]. The detection limits for these biosensors are detection limits as low as 10 ng/mL and enable the earlier detection of cardiac conditions [157]. For the specific diagnostics of myocardial infarction, biosensors targeting cardiac troponin T (cTnT) and cardiac troponin I (cTnI) have impressive detection limits of 10^−3^ ng/mL [158] and have proven to be useful for rapid cardiac assessment in clinical settings [159]. Neurodegenerative diseases have also benefited from the use of SPR biosensor technology and have had systems developed for the detection of biomarkers such as tau-441 for dementia [160]. The biosensors have detection limits of 4.6 × 10^−16^ M and allow for the early diagnosis of conditions like dementia disease [160]. Advanced SPR platforms that have used graphene-based materials have shown exceptional performance at the detection of amyloid-beta oligomers (AβO), a key biomarker of Alzheimer’s disease, and have detection limits as low as 10^−13^ M [161].

**Figure 10:**
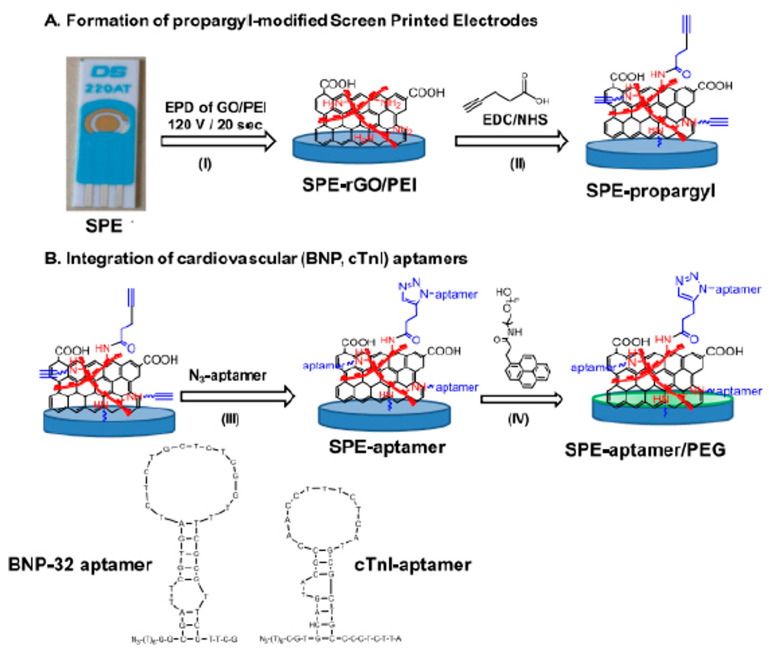
Schematic of a graphene-enhanced biosensor for cardiac biomarker detection. (**A**) Functionalize a gold screen-printed electrode (SPE) using electrophoretic deposition of graphene oxide/polyethylenimine (GO/PEI), followed by EDC/NHS activation. (**B**) Site-specific attachment of aptamers for BNP-32 and cTnI detection through analyte-induced changes in the sensor response. (I) Electrophoretic deposition of GO/PEI onto SPE. (II) Propargyl functionalization via EDC/NHS chemistry (III) click conjugation of azide-modified aptamers for BNP-32 or cTnI (IV) PEG modification for antifouling surface treatment [144].

Figure 10 demonstrates a practical implementation of graphene-based biosensing for cardiovascular disease diagnostics, showcasing the fabrication and functionalization of screen-printed electrodes (SPEs) for aptamer-based detection. In Part A, a multilayer graphene oxide–polyethyleneimine (GO/PEI) interface is electrochemically deposited onto the SPE and chemically activated using EDC/NHS coupling, followed by propargyl functionalization. Part B highlights the selective integration of disease-specific aptamers BNP-32 and cardiac troponin I (cTnI) onto the electrode surface via click chemistry. The incorporation of PEG enhances biocompatibility and reduces nonspecific interactions. This strategy illustrates how graphene-modified electrochemical sensors enable targeted, label-free, and real-time detection of clinically relevant cardiovascular biomarkers, aligning with the broader goal of developing sensitive, point-of-care diagnostic tools.

### 4.3. Pathogen and Virus Detection

Graphene-enhanced SPR biosensors have demonstrated exceptional capability in the detection of various pathogens, including bacteria like Mycobacterium tuberculosis (TB) and viruses such as SARS-CoV-2 and influenza [162]. The integration of graphene with SPR technology creates sensing platforms that enable the identification of pathogens at extremely low concentrations, making them valuable tools for the early diagnosis of infectious diseases [145].

For the detection of tuberculosis, a label-free reflective index graphene-based sensor using a machine learning approach has been developed that identifies the presence of Mycobacterium tuberculosis bacteria with high sensitivity [163]. Additionally, a graphene-based field-effect transistor (GFET) biosensor has also been designed for detecting the MPT64 protein of the pathogen Mycobacterium tuberculosis with exceptional sensitivity as a tool for the detection of TB diagnosis [143]. Such advanced sensing tools overcome the limitations of traditional methods of TB detection by offering rapid and accurate diagnosis without extensive sample preparation [143]. In the context of viral detection, graphene-based enhanced SPR biosensors have shown results for SARS-CoV-2 identification [163]. It has been shown by evidence that the presence of graphene in the SPR biosensors sharpens the resonance dip and induces a slight shift of the angle of the SPR, improving the detection sensitivity [164]. The functionalization of the graphene surface with specific recognition elements further enhances the biosensor’s specificity for SARS-CoV-2 detection [97]. The SPR biosensors designed for SARS-CoV-2 detection have demonstrated exceptional performance, achieving angular sensitivities as high as 371.67°/RIU, where one RIU represents a unit change in the refractive index of the sensing medium due to viral biomolecular binding, indicating exceptional performance in detecting the virus [165]. For the detection of the influenza virus, a simple fluorometric device was designed using the application of graphene oxide (GO) for the identification of influenza subtype viral genes [166]. The system employs a fluorescent DNA probe that reacts with graphene oxide to enable sensitive detection of influenza viral RNA [166]. Additionally, graphene metasurfaces-based SPR biosensors designed for the THz regime have shown promise for virus detection, including influenza [167]. These advanced biosensors can detect changes in the concentration of viral biomolecules, providing a sensitive platform for influenza identification [145].

### 4.4. Multiplexing and Point-of-Care (PoC)

The integration of graphene-gold hybrid SPR biosensors with microfluidic systems has enabled significant advancements in multiplexed detection and point-of-care (PoC) applications [168]. These integrated systems allow for the simultaneous detection of multiple biomarkers or pathogens in a single assay, enhancing diagnostic efficiency and reducing analysis time [169]. The combination of microfluidics with SPR technology facilitates the handling of small sample volumes, minimizes reagent consumption, and enables automated sample processing, making these systems ideal for PoC applications [170]. Graphene’s versatile functionalization potential makes it particularly valuable for multiplexed biosensing applications [170]. The material can be utilized across a variety of single or multiplexed point-of-care biosensing devices, offering high-sensitivity detection with minimal sample preparation [171]. Graphene-based PoC diagnostics eliminate the need for signal amplification and require minimal sample preparation to provide accurate results, significantly reducing time-to-result from days or hours to minutes. This rapid turnaround is crucial in a clinical setting where timely diagnosis can significantly impact patient outcomes [172]. Recent developments include a portable fiber-optic surface plasmon resonance (FO-SPR) device enhanced with graphene for real-time detection of infectious pathogens. This system combines the advantages of fiber-optic technology with graphene-enhanced sensitivity, creating a compact platform suitable for field applications [173,174]. Such portable SPR devices enable real-time monitoring of biomarker levels or pathogen presence without requiring sophisticated laboratory equipment, making them valuable tools for remote healthcare settings [175,176]. The application of graphene-gold hybrid SPR biosensors in multiplexed systems has been demonstrated for various clinical applications, including the simultaneous detection of cancer biomarkers such as CEA and AFP. These multiplexed systems show excellent sensitivity, specificity, and minimal cross-reactivity between different target biomarkers, with detection limits in the picogram per milliliter range. Additionally, researchers have developed a 32-plex graphene biosensor chip that is 100 times more sensitive than conventional lateral flow devices, providing results within 5 min for applications like biotoxin detection [170].

### 4.5. Comparative Performance Analysis

A comprehensive comparison of various SPR biosensor configurations reveals significant sensitivity, detection limits, and specificity variations under different designs and target applications [144]. Sensitivity, expressed in degrees per RIU (°/RIU) or nanometers per RIU (nm/RIU), is a fundamental parameter for the assessment of the performance of the SPR biosensors [176]. It was reported that the peak sensitivity of a monolayer graphene-enhanced SPR biosensor based on a silver layer was up to 300.26°/RIU, 119% or 200% higher than the conventional SPR biosensors without graphene enhancement [176]. For the detection of disease biomarkers, SPR biosensors have been applied to various biological species relevant to human health, including nucleic acids, proteins, viruses, bacteria, and circulating tumor cells [177]. The performance of these biosensors varies depending on the target biomarker and the specific configuration employed [178]. For example, gold electrode-based biosensors focused on the targeting of phosphoglucose isomerase from rabbit muscle for cancer detection in human plasma can detect limits as low as 6.6 × 10^−15^ M [179]. Similarly, the cardiac markers cTnT and cTnI biosensors can detect limits of 10^−3^ ng/mL for the detection of myocardial infarction [151]. The integration of graphene with various materials and configurations has yielded impressive performance improvements in SPR biosensors [180,181]. A graphene/CaF2 multilayer-based laba el-free SPR sensor has demonstrated sensitivity of up to 9000 nm/RIU for first-order resonance and 38,000 nm/RIU for second-order resonance [145]. The sensor detects material concentration changes of gases and biomolecules at a detection accuracy of 0.001 refractive index units (RIU). The sensor capabilities extend to detecting cancers with sensitivity in the range of 6000–7000 nm/RIU and various viruses with sensitivities up to 38,000 nm/RIU [145]. A comparative study evaluating noble metal-graphene-WS_2_ hybrid SPR structures revealed that Ag/Graphene/WS_2_ achieved the highest sensitivity of 240.7°/RIU, followed by Au (200.6°/RIU), Cu (180.4°/RIU), and Al (168.1°/RIU) configurations at 632.8 nm. While Cu and Al-based sensors offer cost-effective platforms, they exhibit lower sensitivity and higher susceptibility to oxidation, confirming that Ag and Au remain optimal choices for achieving high-performance, stable SPR biosensors [182]. A graphene-MoS_2_-Cu hybrid SPR biosensor achieved a resonance angle shift of 76.94°, outperforming both MoS_2_-Cu (74.82°) and graphene-Cu (73.56°) configurations. This enhancement highlights the synergistic optical properties of the graphene-TMDC interface and confirms the superior sensitivity of graphene-based hybrid multilayer systems over conventional or single-material designs [183]. Complementing these findings, recent innovations in metal–dielectric–graphene heterostructures using Al_2_O_3_, ZrO_2_, and Si_3_N_4_ have demonstrated spectral sensitivities exceeding 30,000 nm/RIU, attributed to strong field confinement at the dielectric–graphene interface [184]. Similarly, metal-ITO-graphene/TMDC hybrid multilayers have shown enhanced phase-sensitive SPR performance, while optically active nanomaterials, especially carbon-based and composite systems, are increasingly employed in fiber-optic sensing to improve response time, biocompatibility, and detection limits [185,186].

Table 4 summarizes key advancements in biosensing platforms targeting disease biomarkers, highlighting the use of various working electrodes, bioreceptors, and materials, including graphene-based hybrids, for enhanced detection sensitivity. The listed examples span applications in cancer, cardiovascular, neurodegenerative, inflammatory, and viral diseases, with detection limits reaching as low as the femtomolar range. This comparative overview emphasizes the diagnostic potential of graphene-integrated plasmonic and electrochemical sensors in clinical settings.

The integration of graphene and other nanomaterials has consistently improved sensitivity and lowered detection limits across different applications [145]. For SARS-CoV-2 detection specifically, SPR biosensors have demonstrated exceptional sensitivity, with some configurations achieving sensitivities of 371.67°/RIU. These biosensors can detect minute amounts of viral proteins or antibodies, making them valuable tools for COVID-19 diagnosis [187]. Similarly, for cancer biomarker detection, graphene-enhanced SPR biosensors have achieved detection limits as low as 2.3 × 10^−15^ M for microRNA-21, a biomarker for breast cancer [188].

**Figure 11:**
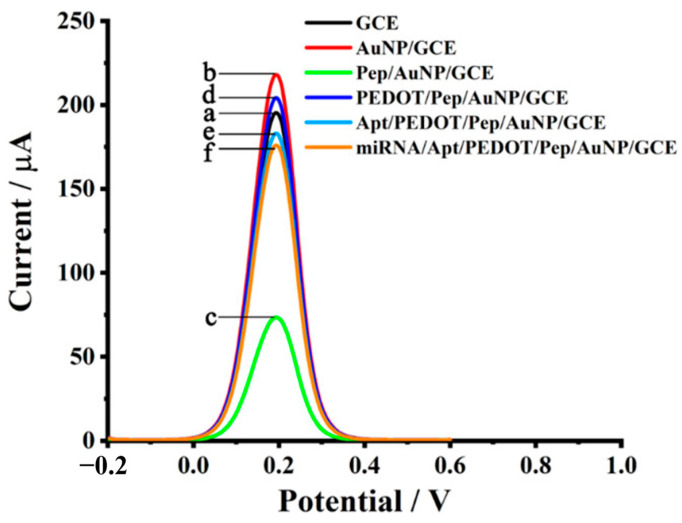
Electrochemical detection of miRNA-21 using various modified electrode configurations. The DPV curves demonstrate signal enhancement from bare DCE through successive functionalization, with the final configuration miRNA-21/Apt/PEDOT/Pep/AuNP/GCE showing the highest current response, indicating superior sensitivity. This graphene-integrated platform enables efficient and label-free detection of microRNA biomarkers relevant to cancer diagnostics [188].

Figure 11 demonstrates the electrochemical performance of a stepwise modified biosensor platform based on miRNA-functionalized aptamer/PEDOT/Pep/AuNP composites. The differential peak currents reflect each modification stage, with the highest current observed for the fully assembled miRNA/Apt/PEDOT/Pep/AuNP/GCE system, indicating enhanced electron transfer and target detection capability. This comparative analysis underscores the cumulative impact of each component, especially the synergistic effect of AuNPs and conductive PEDOT, on improving biosensor sensitivity

## 5. Challenges and Future Perspectives

The integration of graphene plasmonic nanoantennas has demonstrated significant promise in advancing the sensitivity, selectivity, and tunability of surface plasmon resonance (SPR)-based biosensors. However, the transition of these innovations from proof-of-concept designs to real-world deployment remains constrained by several critical technical and practical challenges.

A primary limitation lies in the reproducibility of high-quality graphene and plasmonic metal films, paramount to achieving consistent SPR responses. Variations in thickness, grain boundaries, and surface roughness directly impact plasmonic resonance behavior, leading to inconsistent sensor performance. Moreover, the scalable and cost-effective fabrication methods, such as large-area CVD-grown graphene and template-free nanostructuring, are still under refinement to maintain structural integrity while preserving optical quality [189], while preserving optical and plasmonic characteristics remains a substantial bottleneck in sensor manufacturing [190,191,192]. Moreover, the integration of graphene with diverse materials such as metal oxides, sulfides, or perovskites has emerged as a route to overcome the limitations of noble-metal-only designs. These heterostructures offer enhanced stability, broader plasmon tunability, and improved biocompatibility [141]. The performance of the SPR sensor is highly dependent on the efficient and reproducible immobilization of biomolecules onto the graphene or gold surface. However, controlling surface chemistry across large batches presents challenges. Functionalization methods must ensure specificity, prevent nonspecific adsorption, and remain stable over time and under physiological conditions [193].

The biofunctionalization of graphene and gold surfaces, critical for selective analyte detection, suffers from issues like nano-specification binding, inconsistent molecular orientation, and long-term instability. The π-π stacking capabilities of graphene, while beneficial, can complicate the control of the orientation and density of the capture molecules [194]. Furthermore, in complex media like blood serum or saliva, signal interference, fouling, and low signal-to-noise ratios continue to reduce assay performance. Although graphene enhances local electromagnetic fields and signal amplification, maintaining sensitivity and selectivity in these environments remains a major challenge. Background interference, nonspecific binding, and fouling can diminish sensor performance, while temperature and pH fluctuations can alter graphene’s electronic properties, affecting the fidelity of signal transduction [195].

To enable point-of-care diagnostics, graphene-enhanced SPR sensors need to be integrated with compact, CMOS-compatible, and wearable platforms. This involves overcoming challenges related to optical alignment, real-time signal coupling, and mechanical stability under miniaturization. Integrating graphene-based SPR sensors with microfluidics, flexible substrates, and on-chip readout systems is a major ongoing direction [138]. Furthermore, the combination of graphene with metamaterials, 3D heterostructures, and smart materials (e.g., phathe se-change compounds like GST) holds potential for creating adaptive, tunable SPR platforms [196,197,198,199]. Looking forward, several transformation research directions are actively redefining the landscape of graphene-based plasmonic biosensing. One avenue involves the application of artificial intelligence (AI) and machine learning (ML) for design optimization and data processing. Algorithms are increasingly being trained to automate the development of nanoantenna geometries, interpret complex biosensing data, and supp-ess background interference in real time, thereby accelerating both design cycles and analytical accuracy by identifying subtle signal patterns and structural-performance relationships [200]. Another innovative approach centers on tunable plasmonics through intercalation, where species such as tin (Sn) or chlorine (Cl) are introduced beneath the graphene layer. This process allows dynamic modulation of graphene carrier concentration and dielectric environment, thereby tuning its plasmonic response in the mid-infrared and THz domains [201]. Additionally, as the field pushes towards real-world deployment, there is increasing emphasis on eco-sustainable fabrication. Researchers are exploring green synthesis methods, low-energy processing, and biodegradable substrates to reduce environmental impact while mgintaining device performance [142]. Together, these directions offer a roadmap for the next generation of sensitive, adaptable, and environmewhile also providingasmonic biosensors, adjusting plasmonic ding a dynamic means of adjusting plasmonic behavior. 

In summary, while graphene-enhanced SPR sensors have unlocked new levels of performance in label-free biosensing, realizing their full potential demands interdisciplinary advances in materials engineering, bio-interface chemistry, device integration, and data analytics. Continued development in these areas will accelerate the translation of laboratory-scale innovations into clinical, environmental, and portable diagnostic platforms.

## 6. Conclusions

Significant progress has been made in enhancing surface plasmon resonance (SPR)-based optical biosensors’ sensitivity through the integration of graphene–metal hybrid plasmonic structures. However, key challenges remain before their widespread application can be realized. These include the lack of scalable and reproducible fabrication methods, instability in complex biological environments, and limited clinical validation. Surface functionalization still lacks standardization, and integration with portable systems faces hurdles in optical miniaturization and long-term reliability.

Future efforts should focus on addressing these limitations through interdisciplinary strategies, combining nanomaterials, microfluidics, and AI-driven data processing. Realizing robust, low-cost, and real-time graphene-based SPR platforms will depend on closing these gaps and translating lab-scale advances into clinically and commercially viable technologies.

## Figures and Tables

**Table 1:** Development and comparative analysis of plasmonic nanoantenna designs in SPR biosensing.

Type	Year	Enhancement	Structure	Biosensing Application	Tunable Parameters	Reference	Max Sensitivity (nm/RIU)
Dipole	2003	Moderate	Two metallic nanorods with a gap	SPR, SERS, fluorescence-based detection	Rod length, gap size, dielectric environment	[53]	~1000–2000
Bowtie	2010	Very high	Triangular metallic pair with nanogap	Single-molecule SPR, SERS	Tip sharpness, triangle angle, substrate	[63]	~6000
Hybrid (Au-graphene)	2012	High + Tunable	Au-metal combined with 2D layer	Mid-IR SPR, dynamic biosensing, low-noise SPP	Graphene doping, metal shape, hybrid geometry	[55]	~10,000
Nanoantenna Arrays	2012	Strong coupling effect	Periodic array arrangements	Multiplexed diagnostics, integrated lab-on-chip systems	Array pitch, material combinations, symmetry	[71]	~8000
Slit/ aperture	2023	High (EOT)	Sub-wavelength Slits in metal	Transmission-based SPR, POC detection	Slit width, film thickness, periodicity	[11]	~4000
Graphene Quantum Nanoantenna	2023	Very high (Quantum plasmon)	Hexagonal graphene nano patch on dielectric	Quantum plasmonic, mid-IR detection, integrated chips	Hexagon radius, chemical potential, dielectric substrate	[72]	~12,000
Nanoantenna & nanoradars	2024	Programmable &steerable	Coupled metal–dielectric phase arrays	Reconfigurable biosensing, neural recording	Antenna pitch, phase control, graphene bias	[73]	~15,000

**Table 2:** A comprehensive comparison of their respective advantages and limitations is provided.

Integration Strategy	Advantages	Disadvantages	References
On-chip SPR with nanoantennas	Compact design, real-time sensing, compatible with lab-on-chip systems	Fabrication complexity, optical losses in integrated waveguides	[35]
Kretschmann configuration with nanoarrays	Combining propagating and localized SPR, high sensitivity	Limited reusability, complex prism alignment required	[36]
Waveguide-integrated nanoantennas	Efficient light coupling, small footprint, compatible with silicon photonics	Requires precise refractive index matching, smaller sensing volume	[37]
Graphene-integrated hybrid nanoantennas	Electrically tunable, high sensitivity, enhanced surface chemistry	Material stability, integration challenges, fabrication precision	[38]

**Table 3:** Comparative analysis of graphene vs. metal plasmonic antennas.

Parameter	Conventional metals	Graphene Plasmonic Antennas	Supporting Equation/Figure	Reference
Field confinement	~10× λ reduction	~100× λ reduction	$\lambda p\propto 1/\sqrt{E_{F}}$	[78]
Miniaturization	Limited by λ	5× to 22× smaller	Size and bandwidth diagrams	[79]
Bandwidth	Narrow (freq-fixed)	Ultra-wideband (e.g., 340% for bowtie)	Simulated S-parameters	[54]
Electrical Tunability	None	Continuous via $E_{F}$, $V_{g},chemical\;doping$	Shift in resonance figures	[80]
Sensitivity (nm/RIU)	1000–2000	Up to 7000	Sensor performance plots	[81]
FOM (figure of merit)	<20	60–300 (Hybrids up to 383)	Sensitivity metric figures	[82]
Integration versatility	Moderate	Extensive (polymers, semiconductors, 3D hybrids)	Micrographs, substrate image	[41]
Radiation Efficiency	1–10% (small size)	5–85% (design dependent)	Efficiency simulations	[73]

**Table 4:** Performance comparison of SPR biosensors for disease biomarker detection.

Working Electrode	Bioreceptor	Target Biomarker	Disease Application	Detection Limit	Reference
Gold electrode	D-fructose 6-phosphate	Phosphoglucose isomerase	Cancer in human plasma	6.6 × 10^−15^ M	[148]
Screen-printed gold	Molecularly imprinted polymer	Myoglobin	Cardiovascular disease	2.1 × 10^−3^ ng/mL	[151]
Gold disk	Anti-tau antibody	Tau-441	Dementia	4.6 × 10^−16^ M	[130]
Gold electrode	EGFR antibody	EGFR antigen	Breast cancer	6.9 × 10^−3^ ng/mL	[152]
Gold electrode	TNT-α antibody	TNT-α protein	Inflammation	10^−12^ M	[153]
ZnO	α-cTnT and α-cTnI antibodies	Troponin T& I	Myocardial infarction	10^−3^ ng/mL	[128]
Graphene-gold grating	Thiolated cellular prion protein (PrP^C^) peptide probe	amyloid-beta oligomers (AβO)	Alzheimer’s disease	10^−13^ M–10^−11^ M	[131]
Titanium foil	Co-TiO2 nanotubes	SARS-CoV-2 S-RBD protein	SARS-CoV-2	7 × 10^−10^ M	[154]
Graphene/ CaF2 multilayer	miRNA-21 aptamer	MicroRNA-21	Breast cancer	2.3 × 10^−15^ M	[155]

## Data Availability

This review article does not contain original data. All data discussed are derived from previously published sources, which are appropriately cited in the manuscript.

## References

[1] Sangwan A., Jornet J.M. (2021). Beamforming optical antenna arrays for nano-bio sensing and actuation applications. Nano Commun. Netw..

[2] Gopalan K.K., Paulillo B., Mackenzie D.M., Rodrigo D., Bareza N., Whelan P.R., Shivayogimath A., Pruneri V. (2018). Scalable and tunable periodic graphene nanohole arrays for mid-infrared plasmonics. Nano Lett..

[3] Huang Y.H., Ho H.P., Wu S.Y., Kong S.K. (2012). Detecting phase shifts in surface plasmon resonance: A review. Adv. Opt. Technol..

[4] Sirenko Y.K., Strom S. (2010). Modern Theory of Gratings. Resonant Scattering: Analysis Techniques and Phenomena.

[5] Nguyen H.H., Park J., Kang S., Kim M. (2015). Surface Plasmon Resonance: A Versatile Technique for Biosensor Applications. Sensors.

[6] Malerba M., Alabastri A., Miele E., Zilio P., Patrini M., Bajoni D., Messina G.C., Dipalo M., Toma A., Proietti Zaccaria R. (2015). 3D vertical nanostructures for enhanced infrared plasmonics. Sci. Rep..

[7] Venugopalan P., Kumar S. (2023). Highly Sensitive Plasmonic Sensor with Au Bow Tie Nanoantennas on SiO_2_ Nanopillar Arrays. Chemosensors.

[8] Bludov Y.V., Peres N.M., Vasilevskiy M.I. (2020). Excitation of localized graphene plasmons by a metallic slit. Phys. Rev. B.

[9] Lassiter J.B., Sobhani H., Fan J.A., Kundu J., Capasso F., Nordlander P., Halas N.J. (2010). Fano resonances in plasmonic nanoclusters: Geometrical and chemical tunability. Nano Lett..

[10] Müller R., Bethge J. (2018). Near-field dynamics at a metallic transmission grating with femtosecond illumination: A theoretical study. Phys. Rev. B.

[11] Du G., Yu F., Lu Y., Kai L., Chen C., Yang Q., Hou X., Chen F. (2023). Ultrafast Dynamics of Extraordinary Optical Transmission through Two-Slit Plasmonic Antenna. Nanomaterials.

[12] Wang H., Wang T., Yuan X., Wang Y., Yue X., Wang L., Zhang J., Wang J. (2023). Plasmonic Nanostructure Biosensors: A Review. Sensors.

[13] Li Z., Zhang W., Xing F. (2019). Graphene optical biosensors. Int. J. Mol. Sci..

[14] Rodrigo D., Limaj O., Janner D., Etezadi D., García de Abajo F.J., Pruneri V., Altug H. (2015). Mid-infrared plasmonic biosensing with graphene. Science.

[15] Thongrattanasiri S., Koppens F.H., García de Abajo F.J. (2012). Complete optical absorption in periodically patterned graphene. Phys. Rev. Lett..

[16] Zhou H., Zhang Y., Qiu Y., Wu H., Qin W., Liao Y., Yu Q., Cheng H. (2020). Stretchable piezoelectric energy harvesters and self-powered sensors for wearable and implantable devices. Biosens. Bioelectron..

[17] Gao Z., Shi Y., Li M., Song J., Liu X., Wang X., Yang F. (2021). Tunable extraordinary optical transmission with graphene in terahertz. ACS Omega.

[18] Low T.P. (2014). Avouris, Graphene Plasmonics for Terahertz to Mid-Infrared Applications. ACS Nano.

[19] Daher M.G., Taya S.A., Almawgani A.H.M., Hindi A.T., Colak I., Patel S.K. (2023). Optical biosensor based on surface plasmon resonance nanostructure for the detection of mycobacterium tuberculosis bacteria with ultra-high efficiency and detection accuracy. Plasmonics.

[20] Patel S.K., Alsalman O., Taya S.A., Parmar J. (2023). Skin cancer detection using tunable graphene SPR optical sensor designed using circular ring resonator. Plasmonics.

[21] Yadav A., Kumar S., Kumar A., Sharan P. (2023). Effect of 2-D nanomaterials on sensitivity of plasmonic biosensor for efficient urine glucose detection. Front. Mater..

[22] Homola J. (2008). Surface plasmon resonance sensors for detection of chemical and biological species. Chem. Rev..

[23] Capelli D., Scognamiglio V., Montanari R. (2023). Surface plasmon resonance technology: Recent advances, applications and experimental cases. TrAC Trends Anal. Chem..

[24] Ritchie R.H. (1957). Plasma losses by fast electrons in thin films. Phys. Rev. Lett..

[25] Boardman A.D. (1982). Electromagnetic Surface Modes.

[26] Habib A., Zhu X., Fong S., Yanik A.A. (2020). Active plasmonic nanoantenna: An emerging toolbox from photonics to neuroscience. Nanophotonics.

[27] Xue H., Liu K., Sun C. (2019). Plasmonics for biosensing. Materials.

[28] Liu N., Tang M.L., Hentschel M., Giessen H., Alivisatos A.P. (2011). Nanoantenna-enhanced gas sensing in a single tailored nanofocus. Nat. Mater..

[29] Fischer H., Martin O.J. (2008). Engineering the optical response of plasmonic nanoantennas. Opt. Express.

[30] Koenderink A.F., Alù A., Polman A. (2015). Nanophotonics: Shrinking light-based technology. Science.

[31] Jiang J., Wang X., Li S., Ding F., Li N., Meng S., Li R., Qi J., Liu Q., Liu G.L. (2018). Plasmonic nano-arrays for ultrasensitive bio-sensing. Nanophotonic.

[32] Mehta B., Benkstein K., Semancik S., Mona E.Z. (2016). Gas sensing with bare and graphene-covered optical nano-antenna structures. Sci. Rep..

[33] McPhillips J., Murphy A., Jonsson M.P., Hendren W.R., Atkinson R., Höök F., Zayats A.V., Pollard R.J. (2010). High-performance biosensing using arrays of plasmonic nanotubes. ACS Nano.

[34] Xie J., Ren Z., Wei J., Liu W., Zhou J., Lee C. (2023). Zero-bias long-wave infrared nanoantenna-mediated graphene photodetector for polarimetric and spectroscopic sensing. Adv. Opt. Mater..

[35] Klinghammer S., Uhlig T., Patrovsky F., Bohm M., Schütt J., Pütz N., Baraban L., Eng L.M., Cuniberti G. (2018). Plasmonic biosensor based on vertical arrays of gold nanoantennas. ACS Sens..

[36] Sangwan A., Jornet J.M. (2022). Joint communication and bio-sensing with plasmonic nano-systems to prevent the spread of infectious diseases in the internet of nano-bio things. IEEE J. Sel. Areas Commun..

[37] Ye M., Crozier K.B. (2020). Metasurface with metallic nanoantennas and graphene nanoslits for sensing of protein monolayers and submonolayers. Opt. Express..

[38] Chen K., Guo P., Dao T.D., Shi Q.L., Satoshi I., Tadaaki N., Robert P.H.C. (2017). Protein-functionalized indium-tin oxide nanoantenna arrays for selective infrared biosensing. Adv. Opt. Mater..

[39] Zhou H., Ren Z., Li D., Xu C., Mu X., Lee C. (2023). Dynamic construction of refractive index-dependent vibrations using surface plasmon-phonon polaritons. Nat. Commun..

[40] Li H., Zhang C., Xu H., Yang Q., Luo Z., Li C., Kai L., Meng Y., Zhang J., Liang J. (2024). Microstructured liquid metal based embedded-type sensor array for curved pressure mapping. Adv. Sci..

[41] Calderon J., Alvarez J., Martinez-Pastor J., Hill D. (2014). Bowtie plasmonic nanoantenna arrays for polarimetric optical biosensing. Proc. SPIE Front. Biol. Detect..

[42] Alavirad M., Roy L., Berini P. (2014). Optimization of plasmonic nanodipole antenna arrays for sensing applications. IEEE J. Sel. Top. Quantum Electron..

[43] Kvasnicka P., Homola J. (2008). Optical sensor based on spectroscopy of localized surface plasmons on metallic nanoparticles: Sensitivity considerations. Biointerphases.

[44] Chen H., Kou X., Yang Z., Ni W., Wang J. (2008). Shape- and size-dependent refractive index sensitivity of gold nanoparticles. Langmuir.

[45] Zalyubovskiy S.J., Bogdanova M., Deinega A., Lozovik Y., Pris A.D., An K.H., Hall W.P., Potyrailo R.A. (2012). Theoretical limit of localized surface plasmon resonance sensitivity to local refractive index changes and its comparison to conventional surface plasmon resonance sensor. J. Opt. Soc. Am. A.

[46] Päivänranta B., Merbold H., Giannini R., Büchi L., Gorelick S., David C., Löffler J.F., Feurer T., Ekinci Y. (2011). High Aspect Ratio Plasmonic Nanostructures for Sensing Applications. ACS Nano.

[47] Zhang B., Jornet J.M., Akyildiz I.F., Wu Z.P. (2019). Mutual coupling reduction for ultra-dense multi-band plasmonic nano-antenna arrays using graphene-based frequency selective surface. IEEE Access.

[48] Mamiyev Z., Balayeva N.O., Ghosal C., Zahn D.R.T., Tegenkamp C. (2025). Confinement Induced Strain Effects in Epitaxial Graphene. Carbon.

[49] Mamiyev Z., Tegenkamp C. (2024). Exploring Graphene–Substrate Interactions: Plasmonic Excitation in Sn-Intercalated Epitaxial Graphene. 2D Mater..

[50] Kataria S., Wagner P., Passi V., Lemme M.C. (2021). Chemical Vapor Deposited Graphene: From Synthesis to Applications. arXiv.

[51] Kruskopf M., Pakdehi D.M., Pierz K., Wundrack S., Stosch R., Dziomba T., Götz M., Baringhaus J., Aprojanz J., Tegenkamp C. (2016). Comeback of Epitaxial Graphene for Electronics: Large-Area Growth of Bilayer-Free Graphene on SiC. arXiv.

[52] Cui L., Wang J., Sun M. (2021). Graphene plasmon for optoelectronics. Rev. Phys..

[53] Li K., Stockman M.I., Bergman D.J. (2003). Self-similar chain of metal nanospheres as an efficient nanolens. Phys. Rev. Lett..

[54] Ebbesen T.W., Lezec H.J., Ghaemi H.F., Thio T., Wolff P.A. (1998). Extraordinary optical transmission through sub-wavelength hole arrays. Nature.

[55] Uddin N., Yang Q., Du G., Chen F., Li H., Hou X. (2020). Active Tuning of Hybrid Plasmonics in Graphene-Covered Metallic Nano-trench. Tech. Phys. Lett..

[56] Uddin N., Yang Q., Du G., Chen F., Lankanath D., Li H., Hou X. (2020). Trapping Nanospheres within Graphene-Based Heterogeneous Plasmonic Nano-Trench. J. Opt..

[57] Dash S., Patnaik A., Kaushik B.K. (2019). Performance enhancement of graphene plasmonic nanoantennas for THz communication. IET Microw. Antennas Propag..

[58] Huang S., Song C., Zhang G., Yan H. (2017). Graphene plasmonics: Physics and potential applications. Nanophotonics.

[59] Kumar C., Raghuwanshi S.K., Kumar S. (2024). Comprehensive characterization of a graphene-based plasmonic patch antenna for terahertz applications. Terahertz, RF, Millimeter, and Submillimeter-Wave Technology and Applications XVII.

[60] Rakheja S., Sengupta P., Shakiah S.M. (2020). Design and circuit modeling of graphene plasmonic nanoantennas. IEEE Access.

[61] Ullah Z., Witjaksono G., Nawi I., Tansu N., Irfan Khattak M., Junaid M. (2020). A Review on the Development of Tunable Graphene Nanoantennas for Terahertz Optoelectronic and Plasmonic Applications. Sensors.

[62] Yanase Y., Hiragun T., Ishii K., Kawaguchi T., Yanase T., Kawai M., Sakamoto K., Hide M. (2014). Surface Plasmon Resonance for Cell-Based Clinical Diagnosis. Sensors.

[63] Xu R., Wang D., Zhang H., Xie N., Lu S., Qu K. (2017). Simultaneous Detection of Static and Dynamic Signals by a Flexible Sensor Based on 3D Graphene. Sensors.

[64] Ravindran N., Kumar S., M Y., S R., C A M., S N.T., C K S. (2023). Recent Advances in Surface Plasmon Resonance (SPR) Biosensors for Food Analysis: A Review. Crit. Rev. Food Sci. Nutr..

[65] Tene T., Bellucci S., Arias Arias F., Carrera Almendariz L.S., Flores Huilcapi A.G., Vacacela Gomez C. (2024). Role of Graphene in Surface Plasmon Resonance-Based Biosensors. Sensors.

[66] Negahdari R., Rafiee E., Kordrostami Z. (2023). A Sensitive Biosensor Based on Plasmonic-Graphene Configuration for Detection of COVID-19 Virus. Plasmonics.

[67] Schnell M., García-Etxarri A., Huber A.J., Crozier K., Aizpurua J., Hillenbrand R. (2009). Controlling the near-field oscillations of loaded plasmonic nanoantennas. Nat. Photonics.

[68] Liang J., Yang Q., Zhang C., Tian M., Meng Y., Kai L., Hu T., Chen S., Chen F. (2024). Bioinspired, Anti-Fogging and De-Icing Transparent Surfaces with Flexible Property. Appl. Mater. Today.

[69] Wu C.M., Jian Z.C., Joe S.F., Chang L.B. (2003). High-sensitivity sensor based on surface plasmon resonance and heterodyne interferometry. Sens. Actuators B Chem..

[70] Endo T., Yamamura S., Nagatani N., Morita Y., Takamura Y., Tamiya E. (2005). Localized surface plasmon resonance based optical biosensor using surface modified nanoparticle layer for label-free monitoring of antigen–antibody reaction. Sci. Technol. Adv. Mater..

[71] Benounis M., Jaffrezic N., Martelet C., Dumazet-Bonnamour I., Lamartine R. (2015). High sensitive surface plasmon resonance (SPR) sensor based on modified calix (4) arene self-assembled monolayer for Cadmium ions detection. Mater. Trans..

[72] Fang Z., Wang Y., Liu Z., Schlather A., Ajayan P.M., Koppens F.H., Nordlander P., Halas N.J. (2012). Plasmon-Induced Doping of Graphene. ACS Nano.

[73] Kavitha S., Saxena R.S., Singh A., Kumari K., Aneesh M. (2023). Hexagonal-shaped graphene quantum plasmonic nano-antenna sensor. Sci. Rep..

[74] Matsuo Y., Aoki Y. (2024). Synthetic document images with diverse shadows for deep shadow removal networks. Sensors.

[75] Zhang J., Yang Q., Zhang C., Li H., Zhao H., Chen F. (2024). A review of liquid metal-based flexible electronics achieved by ultrafast lasers. Appl. Mater. Today.

[76] Butt M.A. (2025). Surface Plasmon Resonance-Based Biodetection Systems: Principles, Progress and Applications, A Comprehensive Review. Biosensors.

[77] Zhang S., Qi Y., Tan S.P.H., Bi R., Olivo M. (2023). Olivo, Molecular fingerprint detection using Raman and infrared spectroscopy technologies for cancer detection: A progress review. Biosensors.

[78] Behrouzi K., Wu Z., Lin L., Kante B. (2025). Single plasmonic exceptional point nanoantenna coupled to a photonic integrated circuit sensor. Photon. Res..

[79] Pang H., Cho H.J., Likamwa P.L. (2008). On-chip surface plasmon resonance sensor. J. Nanosci. Nanotechnol..

[80] Shukla N., Chetri P., Boruah R., Gogoi A., Ahmed G.A. (2022). Surface plasmon resonance biosensors based on Kretschmann configuration: Basic instrumentation and applications. Recent Advances in Plasmonic Probes: Theory and Practice.

[81] Ahn H., Song H., Choi J.R., Kim K. (2017). A localized surface plasmon resonance sensor using double-metal-complex nanostructures and a review of recent approaches. Sensors.

[82] Li L., Wu S., Jin M., Zheng Y., Liu Y. (2024). Graphene-enhanced dielectric-metal hybrid structure for high-performance LSPR sensing. Opt. Express.

[83] Zhao Y., Zhu Y. (2015). Graphene-based hybrid films for plasmonic sensing. Nanoscale.

[84] Ansell D. (2015). Graphene for Enhanced Metal Plasmonics. Ph.D. Thesis.

[85] Lin I.T. (2016). Optoelectronic Properties and Plasmonic Devices of Graphene. Ph.D. Thesis.

[86] Fei Z. Nano-plasmonic phenomena in graphene. Proceedings of the 2016 Progress in Electromagnetic Research Symposium (PIERS).

[87] Makeeva G.S. (2024). Electronic Control of Directional Properties of Reconfigurable Plasmonic Graphene-Based Antenna Arrays with Frequency Scanning in the Mid-IR Range. Tech. Phys. Lett..

[88] De Santana E.P., Stock D., Wang Z., Wang K.T., Abadal S., Lemme M., Bolívar P.H. Tunable Plasmonic Graphene Antenna Array for Communications at THz Frequencies. Proceedings of the 2023 48th International Conference on Infrared, Millimeter, and Terahertz Waves (IRMMW-THz).

[89] Suessrneier C., Abadal S., Banszerus L. Analysis of a plasmonic graphene antenna for microelectronic applications. Proceedings of the 2018 43rd International Conference on Infrared, Millimeter, and Terahertz Waves (IRMMW-THz).

[90] Dash S., Patnaik A. Graphene plasmonic bowtie antenna for UWB THz application. Proceedings of the 2018 Twenty Fourth National Conference on Communications (NCC).

[91] Wang X., Meng H., Deng S., Lao C. A nanoscale refractive index sensor based on periodically modulated graphene metamaterial. Proceedings of the 17th International Conference on Optical Communications and Networks (ICOCN2018).

[92] Ogawa S., Fukushima S., Shimatani M. (2020). Graphene plasmonics in sensor applications: A review. Sensors.

[93] Zhang J., Hong Q., Zou J., He Y., Yuan X., Zhu Z., Qin S. (2020). Fano-Resonance in Hybrid Metal-Graphene Metamaterial and Its Application as Mid-Infrared Plasmonic Sensor. Micromachines.

[94] Hosseininejad S.E., Alarcón E., Komjani N., Abadal S., Lemme M.C., Bolívar P.H., Cabellos-Aparicio A. Surveying of pure and hybrid plasmonic structures based on graphene for terahertz antenna. Proceedings of the 3rd ACM International Conference on Nanoscale Computing and Communication.

[95] da Silva W.C., Paiva R.R., de Sousa G.T., da Costa K.Q. Graphene-based terahertz plasmonic sensor. Proceedings of the 2019 SBMO/IEEE MTT-S International Microwave and Optoelectronics Conference (IMOC).

[96] Huang Y., Zhong S., Yao H., Cui D. (2017). Tunable terahertz plasmonic sensor based on graphene/insulator stacks. IEEE Photonics J..

[97] Ma T., Yao B., Zheng Z., Liu Z., Ma W., Chen M., Ren W. (2022). Engineering graphene grain boundaries for plasmonic multi-excitation and hotspots. ACS Nano.

[98] Shameli M.A., Safian R. Waveguide-fed graphene-based hybrid plasmonic patch antenna. Proceedings of the 2017 Iranian Conference on Electrical Engineering (ICEE).

[99] Rodriguez-Lopez P., Antezza M. (2024). Graphene conductivity: Kubo model versus QFT-based model. arXiv.

[100] Ijeomah G., Samsuri F., Zawawi M.A.M., Obite F. (2017). Carbon Nanotube-Graphene hybrid: Recent Synthesis Methodologies and Applications. Int. J. Eng. Technol. Sci..

[101] Azevedo J.D., Queirós T., Camarneiro F., Lopes M.J., Freitas J., Purwidyantri A., Prakash P.S., Chandrasekhar S., Schmidt T.-L., Alpuim P. (2025). Hybrid DNA Origami–Graphene Platform for Electrically-Gated Nanoscale Motion. Adv. Mater. Interfaces.

[102] Chen C.S., Li M.H., Lin S.H., Chiu Y.S., Chen H., Wang D.Y., Han J. (2024). Broadband Photo/Gas Dual Sensors Enabled by ZnO Nanorod/Graphene Hybrid Structures. IEEE Sens. J..

[103] Ahmad Z., Muljarov E.A., Oh S.S. (2021). Extended frequency range of transverse-electric surface plasmon polaritons in graphene. Phys. Rev. B.

[104] Du G., Lu Y., Lankanath D., Hou X., Chen F. (2021). Theoretical Study on Symmetry-Broken Plasmonic Optical Tweezers for Heterogeneous Noble-Metal-Based Nano-Bowtie Antennas. Nanomaterials.

[105] Dash S., Patnaik A. Dual band reconfigurable plasmonic antenna using bilayer graphene. Proceedings of the 2017 IEEE International Symposium on Antennas and Propagation & USNC/URSI National Radio Science Meeting.

[106] Lin Q., Zhao N., Yao K., Jiang Z., Tian B., Shi P., Chen F. (2018). Ordinary Optical Fiber Sensor for Ultra-High Temperature Measurement Based on Infrared Radiation. Sensors.

[107] Tamagnone M., Perruisseau-Carrier J. (2014). Predicting input impedance and efficiency of graphene reconfigurable dipoles using a simple circuit model. arXiv.

[108] Sharma A., Vishwakarma D.K. Circularly polarized graphene antenna for THz applications. Proceedings of the 2021 IEEE 18th India Council International Conference (INDICON).

[109] Biswas R.V., Arifin F. (2022). Highly Directive Graphene Based Hybrid Plasmonic Nanoantenna for Terahertz Applications. AJSE.

[110] Kavitha S., Mishra S.K., Singh A., Singh S.C. (2024). 4 × 4 graphene nano-antenna array for plasmonic sensing applications. Discov. Appl. Sci..

[111] Alharbi R., Irannejad M., Yavuz M. (2019). A Short Review on the Role of the Metal-Graphene Hybrid Nanostructure in Promoting the Localized Surface Plasmon Resonance Sensor Performance. Sensors.

[112] Chattopadhyay S., Goswami A., Sil M. (2025). Nanobiotechnology: Traditional re-interpreting personalized medicine through targeted therapies and regenerative solutions. Naunyn-Schmiedeberg’s Arch. Pharmacol..

[113] Lankanath Karunasena D.A.D., Du G., Yang Q., Iqbal G., Uddin N., Hou X., Chen F. (2021). Stable Plasmonic Nano-Trapping Using a Hybrid Gold-Graphene V-Trench with an Extremely Deep Potential Well. Opt. Mater. Express.

[114] Du G., Lu Y., Uddin N., Lankanath D., Hou X., Chen F. (2020). Giant Electric Field Enhancement for Plasmonic Imaging via Graphene-Based Nanoslit Optical Superlens. Opt. Mater. Express.

[115] Tamagnone M., Gomez-Diaz J.S., Mosig J.R., Perruisseau-Carrier J. (2012). Reconfigurable terahertz plasmonic antenna concept using a graphene stack. Appl. Phys. Lett..

[116] George W. (2008). Hanson. Dyadic Green’s functions and guided surface waves for a surface conductivity model of graphene. J. Appl. Phys..

[117] Kavitha S., Sairam K.V.S.S., Singh A. (2022). Graphene plasmonic nano-antenna for terahertz communication. SN Appl. Sci..

[118] Iyer G.R., Wang J., Wells G., Guruvenket S., Payne S., Bradley M., Borondics F. (2014). Large-Area, Freestanding, Single-Layer Graphene–Gold: A Hybrid Plasmonic Nanostructure. ACS Nano.

[119] Du Z., Hu B., Cyril P., Liu J., Wang Y. (2017). High sensitivity plasmonic sensor using hybrid structure of graphene stripe combined with gold gap-ring. Mater. Res. Express.

[120] Du G., Lu Y., Dayantha L., Hou X., Chen F. (2022). Molecular-Scale Plasmon Trapping via a Graphene-Hybridized Tip-Substrate System. Materials.

[121] Bai X., Gou X., Zhang J., Liang J., Yang L., Wang S., Hou X., Chen F. (2023). A Review of Smart Superwetting Surfaces Based on Shape-Memory Micro/Nanostructures. Small.

[122] García de Abajo F.J. (2013). Graphene nanophotonics. Science.

[123] Bonaccorso F., Sun Z., Hasan T., Ferrari A.C. (2010). Graphene photonics and optoelectronics. Nat. Photonics.

[124] Kansara V., Patel M. (2025). Exploring the role of graphene-metal hybrid nanomaterials as Raman signal enhancers in early-stage cancer detection. Talanta.

[125] Zhu X., Shi L., Schmidt M.S., Boisen A., Hansen O., Zi J., Xiao S., Mortensen N.A. (2013). Enhanced light-matter interactions in graphene-covered gold nanovoid arrays. Nano Lett..

[126] Kostadinova T., Politakos N., Trajcheva A., Blazevska-Gilev J., Tomovska R. (2021). Effect of graphene characteristics on morphology and performance of composite noble metal-reduced graphene oxide SERS substrate. Molecules.

[127] Zhang C., Li Z., Li H., Yang Q., Wang H., Shan C., Zhang J., Hou X., Chen F. (2022). Femtosecond Laser-Induced Supermetalphobicity for Design and Fabrication of Flexible Tactile Electronic Skin Sensor. ACS Appl. Mater. Interfaces.

[128] Ooi K.J., Tan D.T. (2017). Nonlinear Graphene Plasmonics. Proc. R. Soc. A Math. Phys. Eng. Sci..

[129] Cynthia S., Ahmed R., Islam S., Ali K., Hossain M. (2021). Graphene based hyperbolic metamaterial for tunable mid-infrared biosensing. RSC Adv..

[130] Jiang L., Zeng S., Ouyang Q., Dinh X.Q., Coquet P., Qu J., Yong K.T. (2017). Graphene–TMDC–Graphene Hybrid Plasmonic Metasurface for Enhanced Biosensing: A Theoretical Analysis. Phys. Status Solidi A.

[131] Balci S., Balci O., Kakenov N., Atar F.B., Kocabas C. (2016). Dynamic tuning of plasmon resonance in the visible using graphene. Opt. Lett..

[132] Wang X., Shi Y. (2014). Fabrication Techniques of Graphene Nanostructures.

[133] Alam S.N., Sharma N., Kumar L. (2017). Synthesis of Graphene Oxide (GO) by Modified Hummers Method and Its Thermal Reduction to Obtain Reduced Graphene Oxide (rGO). Graphene.

[134] Gul W., Akbar Shah S.R., Khan A., Ahmad N., Ahmed S., Ain N., Khan R. (2023). Synthesis of graphene oxide (GO) and reduced graphene oxide (rGO) and their application as nano-fillers to improve the physical and mechanical properties of medium density fiberboard. Front. Mater..

[135] Chiu N.F., Chen C.C., Yang C.D., Kao Y.S., Wu W.R. (2018). Enhanced plasmonic biosensors of hybrid gold nanoparticle-graphene oxide-based label-free immunoassay. Nanoscale Res. Lett..

[136] Leem J., Wang M.C., Kang P., Nam S. (2015). Mechanically self-assembled, three-dimensional graphene–gold hybrid nanostructures for advanced nanoplasmonic sensors. Nano Lett..

[137] Feinstein M.D., Almeida E. (2024). Hybridization of graphene-gold plasmons for active control of mid-infrared radiation. Sci. Rep..

[138] Phunklang S., Wongsa F., Krachodnok P. (2024). High-Gain InP-Based Hybrid Plasmonic Nanoantennas Design Using SiO_2_–Graphene–Au Stacked Waveguide. Proceedings of the 2024 International Conference on Power, Energy and Innovations (ICPEI).

[139] Attariabad A., Pourziad A., Bemani M. (2022). A tunable and compact footprint plasmonic metasurface integrated graphene photodetector using modified omega-shaped nanoantennas. Opt. Laser Technol..

[140] Phan A.D., Nguyen C.V., Linh P.T., Huynh T.V., Lam V.D., Le A.T., Wakabayashi K. (2020). Deep Learning for the Inverse Design of Mid-Infrared Graphene Plasmons. Crystals.

[141] Mamiyev Z., Balayeva N.O. (2023). PbS Nanostructures: A Review of Recent Advances. Mater. Today Sustain..

[142] Khani S., Hayati M. (2022). Optical biosensors using plasmonic and photonic crystal band-gap structures for the detection of basal cell cancer. Sci. Rep..

[143] Nurrohman D.T., Chiu N.F. (2021). A review of graphene-based surface plasmon resonance and surface-enhanced raman scattering biosensors: Current status and future prospects. Nanomaterials.

[144] Hanifa Lestari T.F., Irkham I., Pratomo U., Gaffar S., Zakiyyah S.N., Rahmawati I., Hartati Y.W. (2024). Label-free and label-based electrochemical detection of disease biomarker proteins. ADMET DMPK.

[145] Jafari B., Gholizadeh E., Jafari B., Zhoulideh M., Adibnia E., Ghafariasl M., Golmohammadi S. (2023). Highly sensitive label-free biosensor: Graphene/CaF2 multilayer for gas, cancer, virus, and diabetes detection with enhanced quality factor and figure of merit. Sci. Rep..

[146] Yoo S.M., Lee S.Y. (2016). Optical biosensors for the detection of pathogenic microorganisms. Trends Biotechnol..

[147] Khansili N., Rattu G., Krishna P.M. (2018). Label-free optical biosensors for food and biological sensor applications. Sens. Actuators B Chem..

[148] Wang J. (2005). Nanomaterial-based electrochemical biosensors. Analyst.

[149] Harvey D.T. (2016). Analytical Chemistry 2.1.

[150] Swami S., Kayenat F., Wajid S. (2024). SPR biosensing: Cancer diagnosis and biomarkers quantification. Microchem. J..

[151] Atkinson A.J., Colburn W.A., DeGruttola V.G., DeMets D.L., Downing G.J., Zeger S.L., Biomarkers Definitions Working Group (2001). Biomarkers and surrogate endpoints: Preferred definitions and conceptual framework. Clin. Pharmacol. Ther..

[152] Chiu N.F. (2022). The current status and future promise of SPR biosensors. Biosensors.

[153] Michalski A., Cox J., Mann M. (2011). More than 100,000 detectable peptide species elute in single shotgun proteomics runs but the majority is inaccessible to data-dependent LC- MS/MS. J. Proteome Res..

[154] Hewitt S.M., Dear J., Star R.A. (2004). Discovery of protein biomarkers for renal diseases. J. Am. Soc. Nephrol..

[155] Campuzano S., Yánez-Sedeño P., Pingarrón J.M. (2017). Electrochemical bioaffinity sensors for salivary biomarkers detection. TrAC Trends Anal. Chem..

[156] Bellassai N., D’Agata R., Jungbluth V., Spoto G. (2019). Surface plasmon resonance for biomarker detection: Advances in non-invasive cancer diagnosis. Front. Chem..

[157] Kuo Y.C., Lee C.K., Lin C.T. (2018). Improving sensitivity of a miniaturized label-free electrochemical biosensor using zigzag electrodes. Biosens. Bioelectron..

[158] Shanmugam N.R., Muthukumar S., Chaudhry S., Anguiano J., Prasad S. (2017). Ultrasensitive nanostructure sensor arrays on flexible substrates for multiplexed and simultaneous electrochemical detection of a panel of cardiac biomarkers. Biosens. Bioelectron..

[159] Das S., Devireddy R., Gartia M.R. (2023). Surface plasmon resonance (SPR) sensor for cancer biomarker detection. Biosensors.

[160] Li X., Jiang M., Cheng J., Ye M., Zhang W., Jaffrezic-Renault N., Guo Z. (2020). Signal multi-amplified electrochemical biosensor for voltammetric determination of tau-441 protein in biological samples using carbon nanomaterials and gold nanoparticles to hint dementia. Microchim. Acta.

[161] Sun L., Zhong Y., Gui J., Wang X., Zhuang X., Weng J. (2018). A hydrogel biosensor for high selective and sensitive detection of amyloid-beta oligomers. Int. J. Nanomed..

[162] Parmar J., Patel S.K., Katkar V., Natesan A. (2023). Graphene-based refractive index sensor using machine learning for detection of mycobacterium tuberculosis bacteria. IEEE Trans. NanoBiosci..

[163] Taya S.A., Daher M.G., Almawgani A.H., Hindi A.T., Zyoud S.H., Colak I. (2023). Detection of virus SARS-CoV-2 using a surface plasmon resonance device based on BiFeO_3_-graphene layers. Plasmonics.

[164] Tene T., Guevara M., Romero P., Guapi A., Gahramanli L., Vacacela Gomez C. (2024). SARS-CoV-2 detection by surface plasmon resonance biosensors based on graphene-multilayer structures. Front. Phys..

[165] Elsayed H.A., Wekalao J., Mehaney A., Haifa E.A., Mostafa R.A., Ali H., Wail A.Z. (2025). Graphene Metasurfaces Biosensor for COVID-19 Detection in the Infrared Regime. Sci. Rep..

[166] Jeong S., Kim D.M., An S.Y., Kim D.H., Kim D.E. (2018). Fluorometric detection of influenza viral RNA using graphene oxide. Anal. Biochem..

[167] Wekalao J., Patel S.K., Al-zahrani F.A. (2018). Graphene metasurfaces-based surface plasmon resonance biosensor for virus detection with sensitivity enhancement using perovskite materials. Plasmonics.

[168] Kim J. (2021). A Study on the Energy-Harvesting Device with a Magnetic Spring for Improved Durability in High-Speed Trains. Micromachines.

[169] Wallace S., Kartau M., Kakkar T., Davis C., Szemiel A., Samardzhieva I., Karimullah A.S. (2023). Multiplexed biosensing of proteins and virions with disposable plasmonic assays. ACS Sens..

[170] Prattis I., Hui E., Gubeljak P., Schierle G.S.K., Lombardo A., Occhipinti L.G. (2021). Graphene for biosensing applications in point-of-care testing. Trends Biotechnol..

[171] Dhinakaran V., Vigneswari K., Lavanya M., Shree M.V. (2021). Point-of-care applications with graphene in human life. Comprehensive Analytical Chemistry.

[172] Li X., Gong P., Zhao Q., Zhou X., Zhang Y., Zhao Y. (2022). Plug-In Optical Fiber SPR Biosensor for Lung Cancer Gene Detection with Temperature and pH Compensation. Sens. Actuators B Chem..

[173] Peeters B., Safdar S., Daems D., Goos P., Spasic D., Lammertyn J. (2020). Solid-Phase PCR-Amplified DNAzyme Activity for Real-Time FO-SPR Detection of the MCR-2 Gene. Anal. Chem..

[174] Pollet J., Delport F., Janssen K.P., Jans K., Maes G., Pfeiffer H., Lammertyn J. (2009). Fiber optic SPR biosensing of DNA hybridization and DNA–protein interactions. Biosens. Bioelectron..

[175] Jiang S., Qian S., Zhu S., Lu J., Hu Y., Zhang C., Liu S. (2023). A point-of-care testing device utilizing graphene-enhanced fiber optic SPR sensor for real-time detection of infectious pathogens. Biosensors.

[176] Chen S., Lin C. (2019). Sensitivity comparison of graphene-based surface plasmon resonance biosensor with Au, Ag and Cu in the visible region. Mater. Res. Express.

[177] Špringer T., Bocková M., Slabý J., Sohrabi F., Čapková M., Homola J. (2025). Surface plasmon resonance biosensors and their medical applications. Biosens. Bioelectron..

[178] Amontree J., Yan X., DiMarco C.S., Levesque P.L., Adel T., Pack J., Holbrook M., Cupo C., Wang Z., Sun D. (2024). Reproducible Graphene Synthesis by Oxygen-Free Chemical Vapour Deposition. Nature.

[179] Devillers M., Ahmad L., Korri-Youssoufi H., Salmon L. (2017). Carbohydrate-based electrochemical biosensor for detection of a cancer biomarker in human plasma. Biosens. Bioelectron..

[180] Al Mahmud R., Sagor R.H., Khan M.Z.M. (2023). Surface plasmon refractive index biosensors: A review of optical fiber, multilayer 2D material and gratings, and MIM configurations. Opt. Laser Technol..

[181] Aghaei F., Golmohammadi S., Bahador H., Soofi H. (2023). Design of a high-sensitivity graphene-silicon hybrid micro-disk in a square cavity whispering gallery mode biosensor. J. Nanopart. Res..

[182] Keshavarz A., Zamani N. (2020). Performance Enhancement of SPR Biosensors Based on Noble Metals–Graphene–WS_2_. Plasmonics.

[183] Zakirov N., Zhu S., Bruyant A., Lérondel G., Bachelot R., Zeng S. (2022). Sensitivity Enhancement of Hybrid Two-Dimensional Nanomaterials-Based Surface Plasmon Resonance Biosensor. Biosensors.

[184] Kumar S., Wang Z., Zhang W., Liu X., Li M., Li G., Zhang B., Singh R. (2023). Optically Active Nanomaterials and Its Biosensing Applications A Review. Biosensors.

[185] Kravets V.G., Wu F., Yu T., Zheng Z., Andreeva D.V., Grigorenko A.N. (2022). Metal–Dielectric–Graphene Hybrid Heterostructures with Enhanced Surface Plasmon Resonance Sensitivity Based on Amplitude and Phase Measurements. Plasmonics.

[186] Han L., He X., Ge L., Gong Q., Zhang H. (2019). Comprehensive Study of SPR Biosensor Performance Based on Metal–ITO–Graphene/TMDC Hybrid Multilayer. Plasmonics.

[187] Vadlamani B.S., Uppal T., Verma S.C., Misra M. (2020). Functionalized TiO_2_ nanotube-based electrochemical biosensor for rapid detection of SARS-CoV-2. Sensors.

[188] Wang G., Han R., Li Q., Han Y., Luo X. (2020). Electrochemical biosensors capable of detecting biomarkers in human serum with unique long-term antifouling abilities based on designed multifunctional peptides. Analyt. Chem..

[189] Anushkannan N.K., Wekalao J., Patel S.K., Al-Zahrani F.A. (2024). Design of encoded and tunable graphene-gold metasurface-based surface plasmon resonance sensors for glucose detection in the terahertz regime. Plasmonics.

[190] Özdemir Ş.K., Rotter S., Nori F., Yang L. (2019). Parity–time symmetry and exceptional points in photonics. Nat. Mater..

[191] Alaeian H., Dionne J.A. (2014). Parity-time-symmetric plasmonic metamaterials. Phys. Rev A.

[192] Liang G., Huang H., Mohanty A., Shin M.C., Ji X., Carter M.J., Yu N. (2021). Robust, efficient, micrometre-scale phase modulators at visible wavelengths. Nat. Photonics.

[193] Shu X., Li A., Hu G., Wang J., Alù A., Chen L. (2022). Fast encirclement of an exceptional point for highly efficient and compact chiral mode converters. Nat. Commun..

[194] Suvarnaphaet P., Pechprasarn S. (2017). Graphene-based materials for biosensors: A review. Sensors.

[195] Lv W., Liu C., Ma Y., Wang X., Luo J., Ye W. (2019). Multi-hydrogen bond assisted SERS detection of adenine based on multifunctional graphene oxide/poly (diallyldimethyl ammonium chloride)/Ag nanocomposites. Talanta.

[196] Patel S.K., Parmar J., Kosta Y.P., Charola S., Zakaria R.B., Nguyen T.K., Dhasarathan V. (2020). Graphene-based highly sensitive refractive index biosensors using C-shaped metasurface. IEEE Sens. J..

[197] Patel S.K., Parmar J., Ladumor M., Ahmed K., Nguyen T.K., Dhasarathan V. (2020). Numerical simulation of a highly directional optical leaky wave antenna using diamond-shaped graphene perturbations. Appl. Opt..

[198] Shakya A.K., Ramola A., Singh S., Vidyarthi A. (2024). Optimized design of plasmonic biosensor for cancer detection: Core configuration and nobel material coating innovation. Plasmonics..

[199] Shan C., Zhang C., Liang J., Yang Q., Bian H., Yong J., Hou X., Chen F. (2020). Femtosecond Laser Hybrid Fabrication of a 3D Microfluidic Chip for PCR Application. Opt. Express.

[200] Malkiel I., Mrejen M., Nagler A., Arieli U., Wolf L., Suchowski H. (2018). Plasmonic Nanostructure Design and Characterization via Deep Learning. Light Sci. Appl..

[201] Mamiyev Z., Tegenkamp C. (2022). Sn intercalation into the BL/SiC (0001) interface: A detailed SPA-LEED investigation. Surf. Interfaces.

